# 40S Ribosomal protein S6 kinase integrates daylength perception and growth regulation in *Arabidopsis thaliana*

**DOI:** 10.1093/plphys/kiae254

**Published:** 2024-05-03

**Authors:** Marc Boix, Alba Garcia-Rodriguez, Laia Castillo, Bernat Miró, Ferga Hamilton, Sanata Tolak, Adrián Pérez, Carolina Monte-Bello, Camila Caldana, Rossana Henriques

**Affiliations:** Centre for Research in Agricultural Genomics (CRAG) CSIC-IRTA-UAB-UB, Campus UAB, 08193 Barcelona, Spain; Centre for Research in Agricultural Genomics (CRAG) CSIC-IRTA-UAB-UB, Campus UAB, 08193 Barcelona, Spain; Centre for Research in Agricultural Genomics (CRAG) CSIC-IRTA-UAB-UB, Campus UAB, 08193 Barcelona, Spain; Centre for Research in Agricultural Genomics (CRAG) CSIC-IRTA-UAB-UB, Campus UAB, 08193 Barcelona, Spain; School of Biological, Earth and Environmental Sciences, University College Cork, North Mall, Cork T23 N73K, Ireland; Environmental Research Institute, University College Cork, Cork T23 XE10, Ireland; School of Biological, Earth and Environmental Sciences, University College Cork, North Mall, Cork T23 N73K, Ireland; Environmental Research Institute, University College Cork, Cork T23 XE10, Ireland; Centre for Research in Agricultural Genomics (CRAG) CSIC-IRTA-UAB-UB, Campus UAB, 08193 Barcelona, Spain; Max Planck Institute of Molecular Plant Physiology, Potsdam-Golm 14476, Germany; Max Planck Institute of Molecular Plant Physiology, Potsdam-Golm 14476, Germany; Centre for Research in Agricultural Genomics (CRAG) CSIC-IRTA-UAB-UB, Campus UAB, 08193 Barcelona, Spain; School of Biological, Earth and Environmental Sciences, University College Cork, North Mall, Cork T23 N73K, Ireland; Environmental Research Institute, University College Cork, Cork T23 XE10, Ireland

## Abstract

Plant growth occurs via the interconnection of cell growth and proliferation in each organ following specific developmental and environmental cues. Therefore, different photoperiods result in distinct growth patterns due to the integration of light and circadian perception with specific Carbon (C) partitioning strategies. In addition, the TARGET OF RAPAMYCIN (TOR) kinase pathway is an ancestral signaling pathway that integrates nutrient information with translational control and growth regulation. Recent findings in Arabidopsis (*Arabidopsis thaliana*) have shown a mutual connection between the TOR pathway and the circadian clock. However, the mechanistical network underlying this interaction is mostly unknown. Here, we show that the conserved TOR target, the 40S ribosomal protein S6 kinase (S6K) is under circadian and photoperiod regulation both at the transcriptional and post-translational level. Total S6K (S6K1 and S6K2) and TOR-dependent phosphorylated-S6K protein levels were higher during the light period and decreased at dusk especially under short day conditions. Using chemical and genetic approaches, we found that the diel pattern of S6K accumulation results from 26S proteasome-dependent degradation and is altered in mutants lacking the circadian F-box protein ZEITLUPE (ZTL), further strengthening our hypothesis that S6K could incorporate metabolic signals via TOR, which are also under circadian regulation. Moreover, under short days when C/energy levels are limiting, changes in S6K1 protein levels affected starch, sucrose and glucose accumulation and consequently impacted root and rosette growth responses. In summary, we propose that S6K1 constitutes a missing molecular link where day-length perception, nutrient availability and TOR pathway activity converge to coordinate growth responses with environmental conditions.

## Teaser

The Arabidopsis ribosomal protein S6 kinase acts as a signaling hub to ensure that growth responses match available nutrient levels especially under short-day photoperiods.

## Introduction

Plants display distinct growth patterns accordingly to the photoperiod they experience. These responses result from the coordination of cell division in meristems, with cell differentiation and elongation in the different organs. Cell growth requires “de novo” protein synthesis, a process regulated by the TOR (TARGET OF RAPAMYCIN) pathway in eukaryotes (Antikainen et al. 2017). This conserved signaling pathway includes the S6K and it direct target, the 40S ribosomal protein S6 (RPS6) (Henriques et al. 2014; Van Leene et al. 2019). In Arabidopsis (*Arabidopsis thaliana*), the TOR pathway acts as a major signaling hub which includes regulators of auxin signaling (Schepetilnikov and Ryabova 2017, 2018), chloroplast development, autophagy, lipid metabolism and protein synthesis (Van Leene et al. 2019; Scarpin et al. 2020). TOR-mediated growth responses are energy demanding processes which need to be coordinated with the available cellular resources (Dobrenel et al. 2016). Considering that photosynthetic-dependent Carbon (C) availability depends on the light period duration, plants will use different C partitioning strategies to ensure the adequate growth responses are maintained under both short days (SD, 8 h light/16 h dark) and long days (LD, 16 h light/8 h dark) (Mengin et al. 2017). Photosynthates accumulated during the light period will be stored as starch, which will be degraded during the night to provide energy for growth and metabolism, therefore, distinct rhythms of starch synthesis/degradation associate with each day-length (Webb et al. 2019). Starch metabolism is then under light and circadian control to ensure that photoperiod perception is coordinated with resource availability/allocation. This regulation will impact the activity of specific signaling pathways (e.g. TOR) to ensure plant growth occurs in the most favorable conditions (Urrea-Castellanos et al. 2022).

The circadian clock is an intricate molecular network perceiving external cues such as light and temperature to generate daily rhythms of biological processes (Millar 2016; Nohales and Kay 2016; Henriques et al. 2018). The repressilator-like structure of its central oscillator ensures the sequential accumulation of its components. *CCA1* and *LHY* transcripts peak at dawn, PRR/TOC1-family members (PRR9, 7, 5 and TOC1) will be present during the light period until dusk, whereas components of the Evening Complex (ELF3, ELF4, and LUX) accumulate mostly during the night (Henriques et al. 2018). To ensure the robustness of these waving patterns, strict transcriptional, and post-translational regulation is in place. For instance, the precise accumulation of PRR5 and TOC1 proteins depends on their ubiquitination and subsequent degradation, a process that is mediated by the ZEITLUPE (ZTL) family of F-box proteins, particularly ZTL (Más et al. 2003; Kiba et al. 2007; Baudry et al. 2010). This ZTL-dependent regulation is required for accurate timekeeping, since in *ztl* null mutants circadian period is increased (Somers et al. 2004). Besides its role within the clock, ZTL also controls hypocotyl elongation and photoperiod-regulation of flowering time, and was recently shown to interact with a wide range of target proteins, suggesting a broad biological function for this F-box (Zoltowski and Imaizumi 2014; Lee et al. 2018).

Circadian oscillations occur at multiple levels and include post-translational modifications such as phosphorylation. Moreover, diel rhythms of protein synthesis, protein phosphorylation and ribosome loading were identified both in plants and animals (Choudhary et al. 2015; Missra et al. 2015; Robles et al. 2017; Seaton et al. 2018; Krahmer et al. 2022). This regulation would extend to the TOR Pathway since both mammalian TOR and p70S6K behave as diel and circadian-regulated outputs and Arabidopsis RPS6 phosphorylation is under light and circadian control (Khapre et al. 2014b; Enganti et al. 2018; Wu et al. 2019; Krahmer et al. 2022). However, lower TOR activity/levels and/or sugar-depleted conditions resulted in longer periods (Zhang et al. 2019), suggesting a mutual regulatory relationship where circadian information, nutrient availability, and TOR pathway activity are interconnected to ensure optimal growth responses.

Here we show that this regulatory network could include S6K, a direct TOR target that is also under photoperiod and circadian regulation. We found that both *Arabidopsis thaliana* (Arabidopsis) S6K-enconding transcripts (*S6K1* and *S6K2*) are under photoperiod control, but only *S6K1* displays a clear circadian behavior under continuous light (free-running) conditions. In addition, total S6K protein accumulation and phosphorylated-S6K levels oscillate under different photoperiods and this waving pattern depends on 26S proteasome-dependent degradation, especially at dusk, a process that is mediated by ZTL. Disruption of S6K1 protein waveform under SDs (either by depletion or over-accumulation) resulted in altered sugar metabolism and had a detrimental effect in plant growth responses. We hypothesize that within the TOR pathway, S6K1 constitutes a molecular link connecting the diel perception of external cues (e.g. day length duration) with specific cellular processes (e.g. phosphorylation, cell growth) with the ultimate goal of matching plant growth responses with resource availability.

## Results

### *S6K1* transcript levels are regulated by photoperiod and the circadian clock

**Figure 1.**
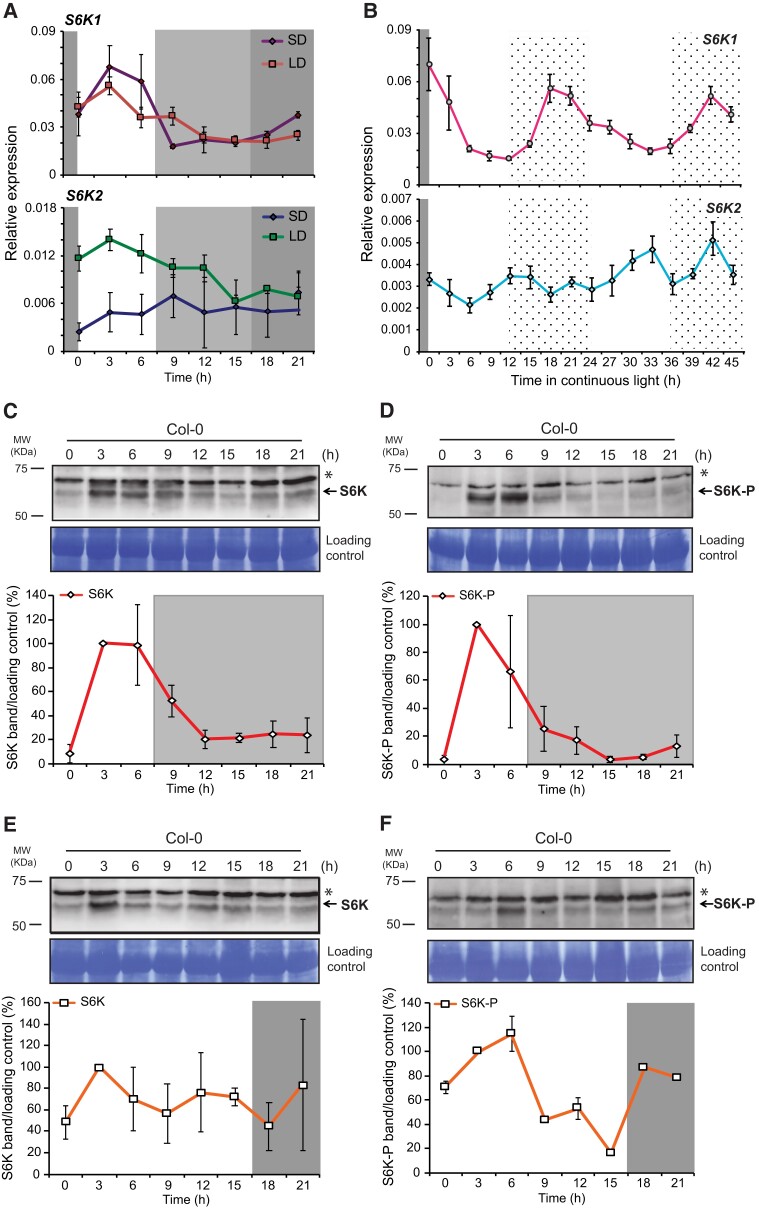
*S6K1* and *S6K2* expression and protein levels are regulated by photoperiod and the circadian clock. *S6K1* and *S6K2* transcript levels were detected by RT-qPCR in 2-week-old wild type seedlings grown under short day (SD, **A)**, long day (LD, **A)** and continuous light conditions (LL, **B)**. Results were analyzed in technical triplicates and normalized to *Actin 2* in two independent biological replicates in **(A)**, in **(B)** the results of one representative biological replicate are shown. Error bars refer to ± standard deviation values. S6K total protein levels determined in 10 day-old seedlings grown under either short day (SD) **(C)** or long day (LD) conditions **(E)**. In parallel, the levels of phosphorylated S6K protein (S6K-P) were detected in the same samples, in SD **(D)** and in LD **(F)**. Loading control corresponds to Coomassie Brilliant Blue staining of the same western membranes used either for anti-S6K or anti-S6K-P detection. Arrows point to S6K or S6K-phosphorylated bands (S6K-P), star indicates an unspecific band. Underneath each western blot is shown the average signal intensity of either the S6K or S6K-P signals after normalization with loading control in two independent biological replicates. Error bars represent ± standard error values. MW represents protein molecular weight marker bands in KDa. Time (h) refers to hours after lights on. Dark gray rectangles correspond to the dark period under LD, whereas lighter gray rectangles correspond to the dark period under SD. Dotted rectangles refer to the subjective night period.

We investigated how photoperiod and circadian conditions would regulate *S6K1* and *S6K2* expression levels. An initial search of available circadian databases (http://diurnal.mocklerlab.org/) revealed that *S6K1* transcript levels displayed a clear oscillatory pattern in wild-type seedlings (WT, Col-0) when grown under SD, LDs and free-running conditions (continuous light for 48 h, LL), an indication of circadian and photoperiod regulation. However, this was not the case for the *S6K2* transcript where we failed to detect a clear waveform under these conditions (Supplementary Fig. S1, A and B). Therefore, we then determined *S6K1* and *S6K2* transcript levels by reverse transcription quantitative PCR (RT-qPCR) in WT seedlings grown under similar conditions (Fig. 1, A and B). We confirmed that *S6K1* transcripts oscillated both under SD and LD, accumulating from ZT0 (ZT—*Zeitgeber* Time) to ZT6, under both photoperiods, being at trough levels around ZT12-ZT15 and gradually increasing towards dawn. Although we did not observe a clear oscillation for the *S6K2* transcript in both photoperiods, it increased in the light period under LDs, and accumulated continuously albeit at lower levels under SDs (Fig. 1A).

When WT seedlings were entrained under 12 h light/12 h dark photoperiods for 2 weeks and then released under free-running conditions (LL), only *S6K1* transcripts displayed a clear oscillation, with a peak at the end of the subjective night and trough at dusk, whereas *S6K2* transcript accumulation was constant (Fig. 1B). These results indicate that photoperiod and the circadian clock modulate *S6K1* expression, whereas *S6K2* does not show such a clear regulation. Considering that S6K is a direct TOR target (Mahfouz et al. 2006; Otterhag et al. 2006; Deprost et al. 2007), we next investigated whether different photoperiods would modulate S6K protein accumulation.

### S6K protein accumulation and phosphorylation is under photoperiod control

To determine the diel pattern of S6K protein accumulation, we firstly used our *s6k1.1*, *s6k2.1,* and *s6k2.2* single insertion mutants to confirm that the commercial anti-S6K antibody (Agrisera Antibodies) recognized both S6K1 and S6K2 proteins (Supplementary Fig. S1C). Nevertheless, we preferably detect S6K1 since *s6k1.1* mutants showed a very faint band, whereas *s6k2* mutants still had a clear, although weaker, band (Supplementary Fig. S1C). We then evaluated the specificity of the anti-S6K-phosphorylated antibody (Agrisera Antibodies) by phosphatase assay (Supplementary Fig. S1D). This antibody recognizes the TOR-dependent phosphorylation site on S6K C-terminal region (S6K-P).

We then investigated how total and phosphorylated S6K protein accumulation was regulated by different photoperiods (Fig. 1, C to F). Under SD conditions, both total S6K and phosphorylated protein levels peaked at ZT3 and then started to decrease at dusk being minimal at ZT15 (Fig. 1, C and D). Under LDs, total S6K was detectable throughout the day with a slight increase at ZT3, whereas S6K-P accumulation peaked at ZT3-ZT6 and then decreased until dusk, increasing again during the night (Fig. 1, E and F). These results showed that total and phosphorylated S6K waveforms peaked in the light period under SDs, and this oscillatory behavior was reduced under LD conditions. These findings lead us to hypothesize that: (i) total S6K protein accumulation is inhibited during the long night under SD conditions; and (ii) TOR complex activity towards S6K (e.g. S6K-P accumulation) is maximal during the light period, especially at ZT3. Considering that we observed a clear oscillation of S6K only under SDs, we then investigated the molecular mechanism behind this regulation.

### The 26S proteasome regulates S6K protein levels under SD conditions

**Figure 2.**
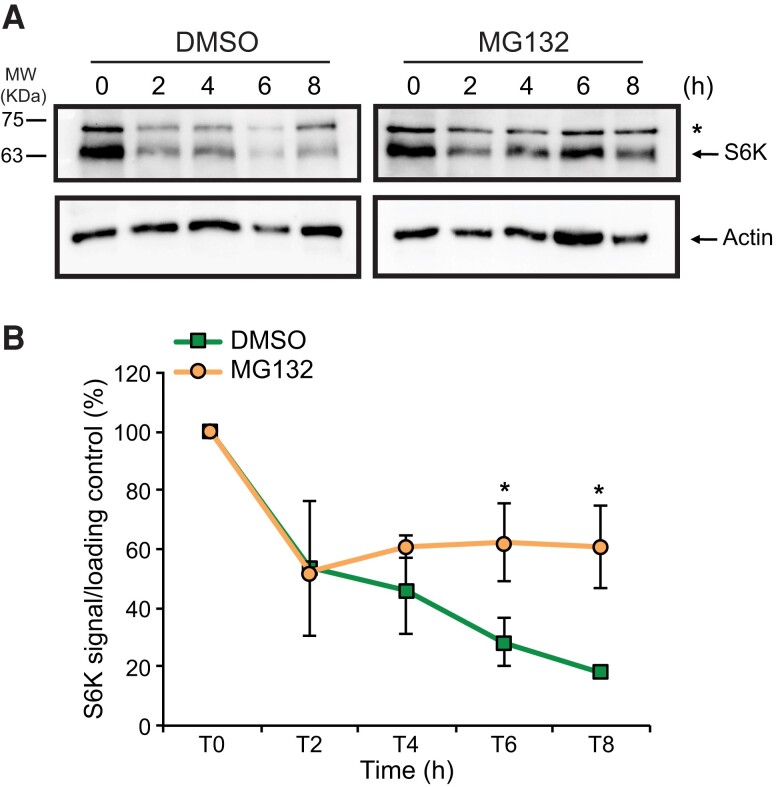
S6K total protein levels are regulated by the 26S proteasome. **A)** S6K total protein levels present in 10 day-old seedlings grown under SDs conditions, either mock-treated (DMSO—Dimethyl sulfoxide; left panels) or incubated with the 26S proteasome inhibitor (MG132; right panels). Seedlings were incubated at ZT3 for the times described (h). The upper panels show total S6K levels, whereas the lower panels reflect the actin levels. Asterisk (*) refers to an unspecific band. MW refers to protein molecular weight marker bands shown in KDa. **B)** Plotted averages of S6K specific band in two independent biological duplicate experiments under the exact same conditions as described in **(A)**. Error bars represent ± standard error values. Details for quantification of results are provided in the Materials and methods section. Statistically significant differences (* indicates *P* < 0.05) were calculated using Student's *t* test for each time point analysed.

Although *S6K1* and *S6K2* transcript levels are mostly stable during the dark period in both photoperiods (Fig. 1A), we detected very low protein levels during the night of SDs, suggesting the existence of degradation mechanism at play. We have previously shown that dark-dependent degradation of PRR5 involved the 26S proteasome and the F-box protein ZTL (Kiba et al. 2007), so we next investigated whether this mechanism could account for S6K accumulation under SDs. We observed that SD-grown WT (Col-0) seedlings treated either with the proteasome inhibitor MG132 or with DMSO (mock) at ZT3 could still maintain 50% of their total S6K amount even after 2 h of treatments. However, after 8 h of incubation MG132-treated seedlings still maintained 50% of S6K total protein levels, whereas mock-treated seedlings had only 20% of their initial S6K amount (Fig. 2), suggesting that S6K protein levels are modulated by the 26S proteasome.

In the ubiquitin-26S proteasome pathway, E3 ligases such as the SCF (SKP1, Cullin, and F-box) complex ubiquitinate and target for degradation-specific substrates (Henriques et al. 2009). Within the circadian clock, the F-box protein ZTL is mostly active at dusk (Kim et al. 2003; Ito et al. 2012; Zoltowski and Imaizumi 2014) coinciding with the observed reduction in S6K total protein levels. Therefore, we investigated whether ZTL could also modulate S6K protein levels, similarly to PRR5 and TOC1 (Más et al. 2003; Kiba et al. 2007).

### The circadian-regulated F-box ZTL modulates S6K stability and half-life

**Figure 3.**
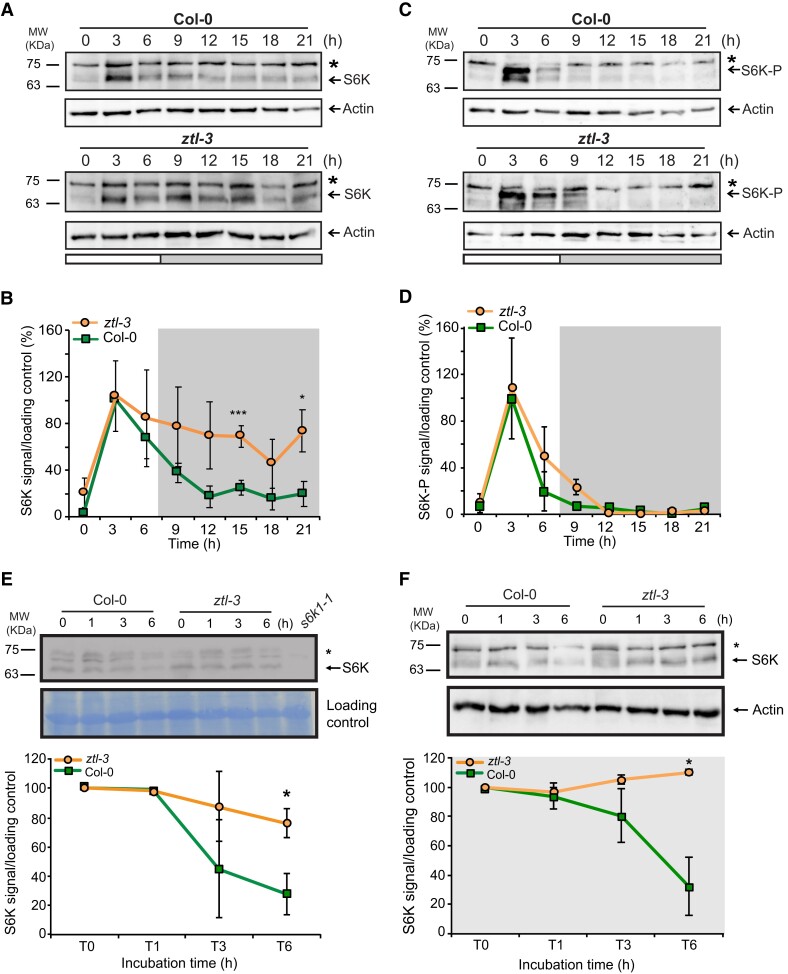
The circadian F-box protein ZTL modulates S6K protein levels and half-life. Total S6K protein accumulates at higher levels and for a longer period of time in *ztl-3* mutant plants than in wild type (WT, Col-0) seedlings grown under SD conditions. **A)** Total S6K and actin protein levels in WT plants (upper panels) and *ztl-3* mutants (lower panels) were detected by western blot analysis using anti-S6K and anti-actin specific antibodies. Arrows point to S6K and actin bands, respectively. Star indicates an unspecific band. **B)** Average S6K signal intensity after normalizing with the corresponding actin signal in three independent biological replicates. **C)** Similarly, phosphorylated S6K is present for a longer period of time in the *ztl-3* mutants (lower panels) when compared with WT (upper panels) seedlings. Loading control was determined by actin levels in the same blots. Arrows point to S6K-P and actin bands, respectively. Star indicates an unspecific band. **D)** Average signal intensity for S6K-P after normalizing with actin loading control in two independent biological replicates. **E)** Upper panels: SD grown 10 day-old WT and *ztl-3* seedlings were incubated at ZT3 with the protein synthesis inhibitor cycloheximide (CHX) and maintained under white light for the indicated hours (h). The *s6k1.1* mutant was used as negative control. Shown is one representative experiment out of two biological replicates. Arrows point to S6K and actin bands, respectively. Star indicates an unspecific band. Lower panel: average S6K signal intensity after normalizing with the loading control in two independent biological replicates. **F)** Upper panels: SD grown 10 day-old WT and *ztl-3* seedlings were incubated at ZT3 with cycloheximide (CHX) and maintained in the dark for the indicated hours (h). Shown one representative experiment out of two biological replicates. Arrows indicate S6K or actin levels, asterisk points to unspecific band. Lower panel: Quantification of the average S6K signal after normalization with the loading control (actin) in two independent biological replicates. Gray rectangle represents the incubation in the dark. Error bars correspond to ± standard error values. MW represents protein molecular weight marker bands shown in KDa. Time refers to hours (h) after lights on; white and gray rectangles indicate the light and dark periods, respectively. Statistically significant differences (* *P* < 0.05, ***P < 0.001) were calculated using Student's *t* test for each time point analyzed.

To address the role of ZTL in modulating S6K levels, we determined total S6K and S6K-P levels in *ztl-3* mutant seedlings lacking a functional ZTL protein (Somers et al. 2004). Firstly, we confirmed that ZTL does not regulate *S6K1* expression, by determining *S6K1* transcript levels in *ztl-3* mutants (Supplementary Fig. S1E). Then we assessed total and S6K-P levels in *ztl-3* seedlings grown under SD conditions. We observed that total S6K protein accumulation increased in *ztl-3* from ZT6 to ZT21, whereas in WT seedlings it was mostly detected during the light period (Fig. 3; Supplementary Figs. S1F and S2). This pattern associated with S6K-P levels which were higher at ZT3 and then gradually disappeared in WT seedlings (Supplementary Fig. S1F). In *ztl-3* mutants, however, S6K-P levels were high from ZT3 to ZT9, possibly due to the stabilization of total S6K. Nevertheless, we observed that TOR-dependent phosphorylation of S6K occurred preferably during the light period, since we failed to detect S6K-P during the night, especially in *ztl-3* mutants that consistently accumulated more total protein during this period (Supplementary Fig. S1F). Although the difference in S6K-P accumulation in *ztl-3* was not statistically significant (Student's *t*-test *P*-value = 0.13 and 0.15, at ZT6 and ZT9, respectively), we found that it was extended for 3 h in this mutant when compared with WT seedlings, even if their peak levels at ZT3 were similar (Fig. 3, A to D; Supplementary Fig. S1F). We then investigated the role of other ZTL family proteins such as FLAVIN-BINDING, KELCH REPEAT, F-BOX 1 (FKF1), and LOV KELCH PROTEIN 2 (LKP2) in regulating total S6K protein accumulation using SD-grown *ztl fkf 1lkp2* triple mutants. We found a similar S6K waveform to that of *ztl-3* single mutants (Supplementary Fig. S1G), an indication that ZTL must be the preferred regulator of S6K accumulation. This was further confirmed in ZTL-overexpressing seedlings (35S::Myc-ZTL) that accumulated very low levels of total S6K protein (Supplementary Fig. S1H).

Next, we determined S6K half-life in 10-day old WT and *ztl-3* seedlings treated with the de novo protein synthesis inhibitor CHX at ZT3 and then kept under light or dark conditions for 6 h. We observed that light-incubated WT and *ztl-3* seedlings had similar S6K protein amounts after 1 h of treatment (Fig. 3E). However, after 6 h of incubation, WT seedlings possessed 50% of their initial S6K amount, whereas in *ztl-3* mutants S6K levels were maintained at 90% (Fig. 3E). Therefore, ZTL depletion under light extended S6K half-life from 3 h to more than 6 h. This effect was stronger in dark-incubated seedlings, where CHX treatment did not greatly affect total S6K protein levels in *ztl-3* mutants, although WT seedlings accumulated only 25% of their initial S6K1 amount after 6 h of incubation (Fig. 3F).

Plants display specific diurnal patterns of growth in rosette leaves and roots, which are under circadian control (Sulpice et al. 2014; Apelt et al. 2017; Urrea-Castellanos et al. 2022). However, little is known about the cellular events underlying these responses. Our results showing that S6K1 protein accumulation and activity is maximal during the light period, provide a mechanistic insight to connect photoperiod perception with the TOR signaling pathway. Therefore, we went to assess the relevance of this process in regulating plant growth responses.

### ZTL-dependent regulation of S6K levels is required for rosette and root growth under short photoperiods

We initially characterized *S6K1* and *S6K2* transcript levels and protein accumulation in cotyledons/young leaves, hypocotyls, and roots of SD-grown 10 day-old WT seedlings (Supplementary Fig. S3, A to C). Our results showed that *S6K1* transcripts levels were higher in young leaves, whereas *S6K2* was mostly detected in hypocotyls (Supplementary Fig. S3, A and B). In parallel, we detected total S6K protein accumulation in young leaves and in roots, although at a lower level (Supplementary Fig. S3C). However, S6K-P was detected in young leaves at ZT6 possibly due to higher total S6K levels in these organs (Supplementary Fig. S3D). Therefore, we hypothesize that S6K accumulation in roots and shoots suggests a role for this kinase in regulating growth responses in these organs.

To further assess the role of S6K in modulating plant growth, we designed two parallel approaches. Firstly, we used the *S6K1p::S6K1g-CFP#*11.6 line where S6K1 is over-expressed under the control of its own promoter and with a C-terminal CFP tag. We confirmed that the CFP tag did not affect S6K1 accumulation nor the ability of endogenous S6Ks or fused S6K1 to phosphorylate the ribosomal protein S6 (RPS6). In addition, both wild type (Col-0) and the S6K1-CFP line show comparable levels of total and phosphorylated S6 protein (Ser237) (Supplementary Fig. S3, E and I). Moreover, we also showed that *S6K1p::S6K1g-CFP#*11.6 was expressed during lateral root formation (Stitz et al. 2023). This line was crossed with the *ztl-3* mutant to genetically address how circadian regulation of S6K protein levels would impact plant growth. We isolated two independent crossed lines, *ztl-3*; *S6K1p::S6K1g-CFP*#11.6 line #2.1.3 and line #3.11.2 (Supplementary Fig. S3, E to G). We showed that S6K1-CFP accumulated throughout the diel cycle under SD conditions, but we failed to observe its degradation during the night period especially from ZT9-15. We hypothesize that the over-accumulation of S6K1-CFP in the dark might not be fully degraded by ZTL. When the F-box was absent, such as in *ztl-3* seedlings, S6K1-CFP was further stabilized and its pattern of accumulation was advanced 3 h (Supplementary Fig. S3, E and F). Secondly, we generated double *ztl-3 s6k1.1* mutants by crossing *ztl-3* with *s6k1.1* and confirmed the absence of both transcripts in two independent lines *ztl-3 s6k1.1* #1.1.1 and #1.1.5 (Supplementary Fig. S3H).

**Figure 4.**
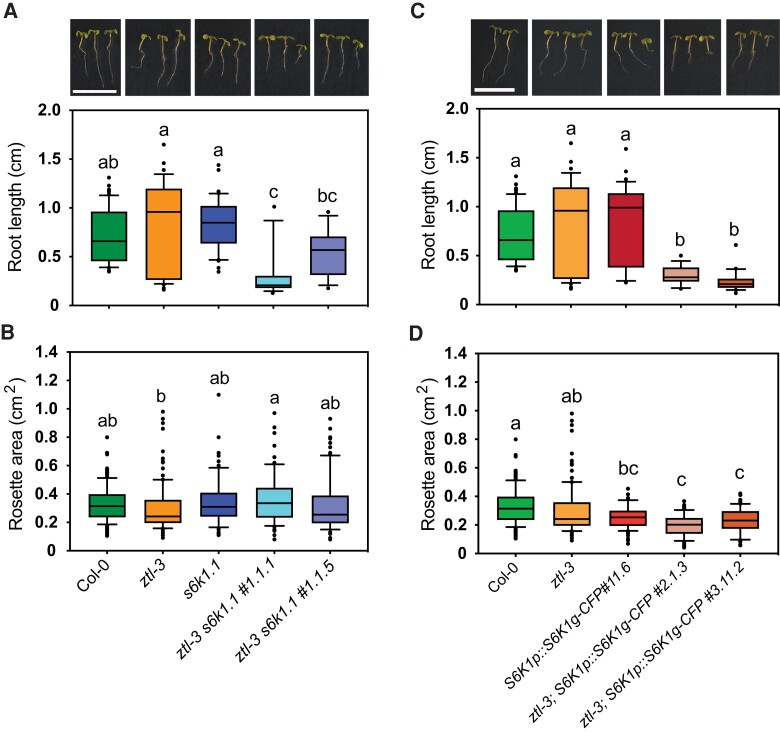
Modulation of *S6K1* levels in *ztl-3* mutants affects plant growth responses. **A)** Upper panel: representative photos of genotypes used to generate *ztl-3 s6k1.1* double mutants. Seedlings were grown in vitro for 10 days under SD conditions. Lower panel: Boxplot analysis of corresponding root length of Col-0 (green, *n* = 84) seedlings, *ztl-3* (orange, *n* = 38), *s6k1.1* (dark blue, *n* = 40) mutants, and *ztl-3 s6k1.1* cross lines #1.1.1 (light blue; *n* = 16) and #1.1.5 (lilac, *n* = 19) in three independent biological replicates. Results shown for Col-0 and *ztl-3* are the same as in **(C)**. However, different representative photos were selected in each upper panel to provide a better overview of these two genotypes. **B)** Boxplot analysis of rosette area of 21-days old SD-grown Col-0 (*n* = 176), *ztl-3* (*n* = 124), *s6k1.1* (*n* = 64), and *ztl-3 s6k1.1* lines #1.1.1 (*n* = 84) and #1.1.5 (*n* = 88) in two independent biological replicates. Colors represent same genotypes as described in **(A)**. **C)** Upper panel: representative photos of genotypes used to generate *ztl-3*; *S6K1p::S6K1g-CFP* lines. Seedlings were grown in vitro for 10 days under SD conditions. Lower panel: Boxplot analysis of corresponding root length of Col-0 (green, *n* = 84) seedlings, *ztl-3* (orange, *n* = 38) mutants, *S6K1p::S6K1g-CFP*#11.6 lines (dark red, *n* = 35) and *ztl-3*; *S6K1p::S6K1g-CFP* cross lines #2.1.3 (beige; *n* = 15) and #3.11.2 (light red, *n* = 21) in three independent biological replicates. **D)** Boxplot analysis of total rosette areas from 21 day-old SD-grown Col-0 (*n* = 176), *ztl-3* mutants (*n* = 124), *S6K1p::S6K1g-CFP*#11.6 seedlings (*n* = 67), and crossed *ztl-3*; *S6K1p::S6K1g-CFP* lines #2.1.3 (*n* = 65) and #3.11.2 (*n* = 66) from three independent biological replicates. Scale bar represents 1 cm in all images of panels **(A)** and **(C)**. Boxplots show the median (black line), and box limits extend from the 25th to the 75th percentiles, whiskers represent the 10th and 90th percentiles, whereas outliers are shown as individual black dots. Different letters correspond to statistically significant differences (*P* < 0.05) determined by One-Way ANOVA followed by Tukey's test (GraphPad Prism).

We then assessed root length and rosette area of SD-grown 10-day and 21-day old developed seedlings, respectively. We observed that *ztl-3*, *s6k1.1,* and *S6K1p::S6K1g-CFP*#11.6 seedlings developed roots of similar length to the wild type (WT, Col-0) (Fig. 4A). *ztl-3 s6k1.1* double mutants had shorter roots than their parents, but in the case of line #1.1.5 those were not significantly different from the WT control. This decrease in root length was stronger in *ztl-3*; *S6K1p::S6K1g-CFP*#11.6 lines #2.13 and #3.11.2 (Fig. 4, A and C), suggesting that, in the absence of proper circadian function, the over-accumulation of S6K1 is more detrimental to root growth than its depletion. Therefore, we went on to characterize average rosette area phenotypes in these lines. We observed that *ztl-3* and *s6k1.1* rosettes were similar to each other and the WT, whereas *S6K1p::S6K1g-CFP*#11.6 rosettes were smaller than WT, but not statistically different from *ztl-3* (Fig. 4, B and D). However, whereas *ztl-3 s6k1.1* double mutants developed rosettes similar to their parents and the WT, *ztl-3*; *S6K1p::S6K1g-CFP*#11.6 rosettes were significantly smaller than *ztl-3* and WT (Fig. 4, B and D). Together, our results indicate that tight regulation of S6K1 levels under SDs is critical to ensure adequate growth responses.

### Changes in S6K1 levels modulate resource accumulation under short photoperiods

**Figure 5.**
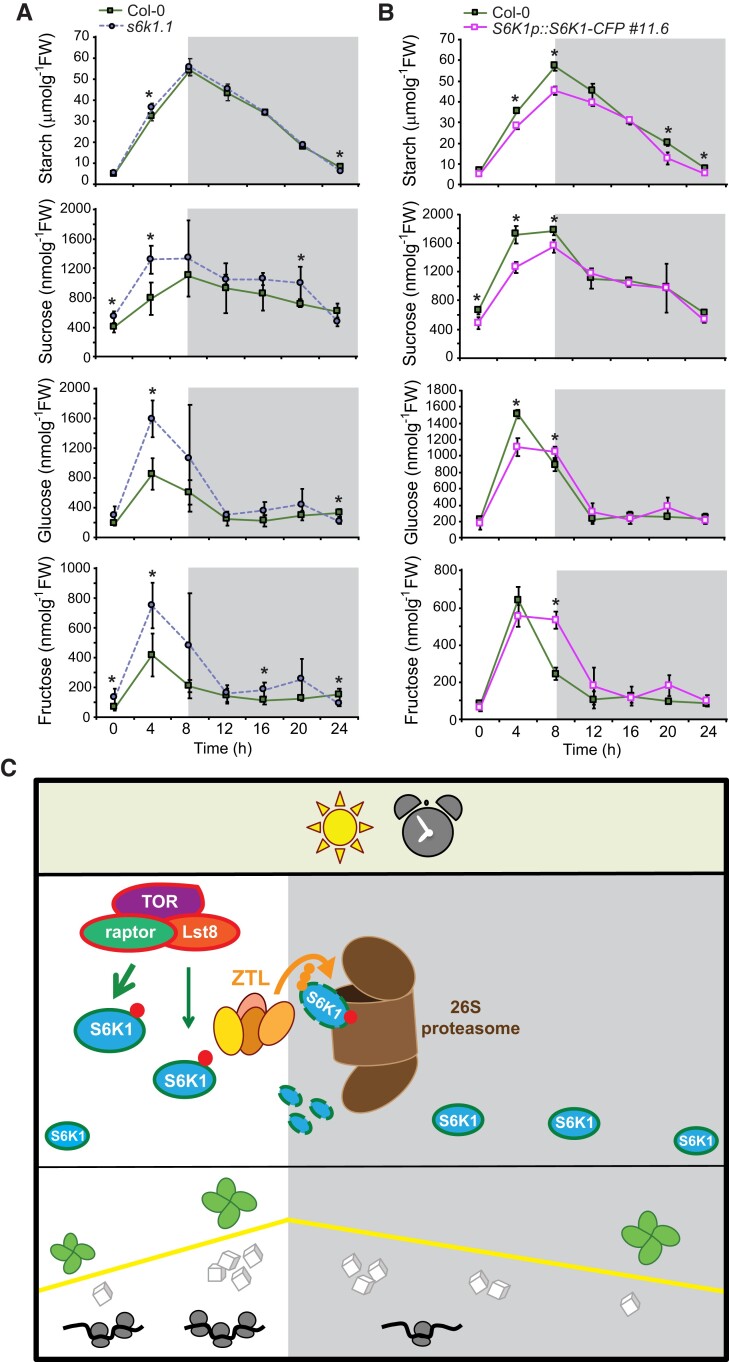
Regulation of S6K1 levels under SD conditions is required for efficient use of resources and adequate growth responses. **A, B)** Variable *S6K1* levels affect diel accumulation of sugars. Top to bottom: accumulation of Starch, Sucrose, Glucose, and Fructose in 30-day old soil-grown seedlings under SD conditions. Pools of three rosettes were analyzed in five biological replicates in Col-0 and *s6k1.1* mutants **(A)** and in three biological replicates in Col-0 and *S6K1p::S6K1g-CFP#11.6* overexpression line **(B)**. The results are the mean ± SD. Statistically significant differences are indicated by asterisks (Student's *t*-test): *P < 0.05, **P < 0.01, and ***P < 0.001. FW refers to fresh weight of analyzed plants (g). **C)** Current working model describing overall impact of circadian (clock drawing) and photoperiod (sun drawing) regulation of S6K1 and consequently, TOR pathway activity. S6K1 protein levels start to increase at dawn and will be maximal between ZT3-6 when TOR-dependent phosphorylated S6K also accumulates. At dusk, ZTL will mediate S6K1 proteasome-dependent degradation. S6K1 protein levels will then be minimal during the long dark period. This regulatory network will allow the coordination between C/energy use and TOR pathway activity (depicted as translational capacity) to ensure that plant growth responses are perfectly matched with the available resources especially during the long night. White cubes show available C for growth and its pattern of accumulation is depicted by the yellow line, green rosettes represent Arabidopsis, and gray circles connected to the black lines refer to ribosomes associated to mRNAs (to depict translational capacity). White and gray rectangles indicate the light and dark periods, respectively.

We hypothesize that photoperiod-dependent regulation of S6K1 is necessary to coordinate TOR pathway activity and growth with resource availability, especially under shorter days when there are less hours available for photosynthesis (Urrea-Castellanos et al. 2022). Therefore, we determined starch, sucrose, glucose, and fructose levels in *s6k1.1* and *S6K1p::S6K1g-CFP*#11.6 rosettes of soil-grown seedlings and compared them with WT plants grown under similar conditions (Fig. 5). We observed that *s6k1.1* mutants accumulated similar levels of starch to WT seedlings with the exception of ZT4 when there was a slight increase in the mutants. Interestingly, this is the period when S6K total and phosphorylated protein levels are maximal (Figs. 1 and3). Similarly, sucrose, glucose, and fructose levels were also elevated in these mutants at a similar time of the day (Fig. 5A). We also observed that *s6k1.1* plants accumulated higher sugar content during the light period, but there were not many differences during the night. In a parallel experiment, we compared WT and *S6K1p::S6K1g-CFP*#11.6 seedlings and found an opposite response, where the latter accumulated less starch during the light period (ZT4-8) and at the end of the night (ZT20-24). Sucrose levels were also lower in *S6K1p::S6K1g-CFP*#11.6 seedlings during the light period (ZT0-ZT8), whereas glucose and fructose levels were lower at ZT4 but they increased at ZT8, although no differences were found during the dark period (Fig. 5B). These findings would suggest that over-accumulation of S6K1 could affect resource allocation or result in higher energy costs than the plant can sustain, with the consequent impact in overall rosette growth.

This interconnection between S6K levels and C assimilation/allocation further strengthens our hypothesis that photoperiod-dependent regulation of the TOR pathway is important to ensure the proper use of available resources. Considering that starch metabolism is also associated with photoperiod perception and under circadian regulation (Henriques et al. 2018), we propose a mechanism where the clock could match S6K1 accumulation and activity with metabolic status in order to ensure the adequate growth responses for specific photoperiods.

## Discussion

### S6K is a molecular link connecting the TOR pathway with photoperiod perception

Plants are able to integrate external cues (e.g. duration of light and dark periods) with specific internal processes, to ensure that growth responses, and developmental transitions perfectly match with their environmental conditions. Therefore, distinct photoperiods associate with specific C partitioning strategies, which are reflected in different growth rhythms (Urrea-Castellanos et al. 2022). Several lines of evidence suggest a role for the TOR pathway in the regulation of these photoperiod-dependent growth responses (Salem et al. 2018; Moreau et al. 2012) which would also involve a mutual connection with the circadian clock. On one hand, TOR depletion or inhibition resulted in longer photoperiods (Zhang et al. 2019); and on the other, core clock components also affected TOR mRNA degradation, even if indirectly, via TZF1, a RNA-binding protein (Li et al. 2019; Wang et al. 2020). This regulation also extends to RPS6 phosphorylation which is both under light and clock regulation (Enganti et al. 2018), and could constitute a sensing mechanism for seasonal perception, providing plants with a predictive strategy to anticipate day length changes (Panchy et al. 2020). Our findings further characterize this molecular link by showing that S6K protein accumulation and activity are under circadian regulation.

We found that the rhythmic accumulation of S6K resulted from a combined transcriptional and post-translational regulation (Fig. 1). Under SDs, S6K protein levels are higher during the day, and minimal at dusk and night, when specific circadian F-box proteins accumulate (ZT7 to ZT19) (Kim et al. 2003; Lee et al. 2019). Different observations lead us to propose that ZTL is a critical mediator of 26S proteasome-dependent degradation of S6K. First, 26S proteasome inhibition promoted S6K accumulation (Fig. 2). Second, total S6K levels were equally stabilized in *ztl-3* and *ztlfkf1lkp2* triple mutants (Fig. 3, Supplementary Fig. S1). Third, S6K half-life was increased in *ztl-3* mutants (Fig. 3). Although we could not detect a stable in vivo interaction between S6K and ZTL, this could be due to the low levels of S6K accumulation in the Myc-ZTL seedlings used in these assays, or to the possible transient nature of such interaction (Supplementary Fig. S1). Interestingly, RPS6 was identified as a ZTL interactor (Lee et al. 2018), reinforcing our hypothesis of a direct connection between S6K and this F-box protein.

*ztl-3* mutants accumulated phosphorylated S6K from ZT3 to ZT9, 3 h longer than WT seedlings, suggesting that TOR-dependent phosphorylation could also signal for its proteasome-dependent degradation. A similar mechanism was identified in *Saccharomyces pombe* where TOR phosphorylation of the meiosis regulator Mei2p preceded ubiquitination-mediated turnover (Otsubo et al. 2014). In *Arabidopsis*, the *Mei2p* homolog (AML1) could bind Raptor in a yeast two-hybrid assay (Anderson and Hanson 2005), and was recently identified as a TOR-regulated phosphoprotein. Moreover, S6K could associate with Rpn2/Psmd1 (Regulatory Particle non-triple-A ATPases), a subunit of the 26S proteasome regulatory particle (Book et al. 2010; Van Leene et al. 2019). Possibly this connection between TOR phosphorylation and the protein degradation machinery could ensure timely disposal of pathway components after activation, similarly to what was reported in animals (Saxton and Sabatini 2017). Interestingly, modeling approaches have shown that phosphorylation events associated with ubiquitination and degradation processes help reduce the biosynthetic costs of rhythmic accumulation of circadian proteins (Lim et al. 2021), which might explain why 65% of oscillating proteins are phosphoproteins (Choudhary et al. 2016). Furthermore, the identification of a phospho-dawn mechanism in plants, which was suggested to include S6K, further strengthens the connection between phosphorylation-based signaling (e.g. TOR pathway) and the circadian clock (Krahmer et al. 2022).

### S6K1 regulates photoperiod-dependent growth responses and resource availability

We hypothesize that photoperiod regulation of S6K accumulation and activity would ensure the correct timing of growth responses. Under SDs, rosette growth rates of *Arabidopsis* wild-type seedlings peak at ZT2-ZT4 (Dornbusch et al. 2014; Apelt et al. 2017) overlapping with the period where we observed: (i) an increase in *S6K1* transcript levels, (ii) greater total S6K protein accumulation and, (iii) higher TOR-dependent S6K-phosphorylation (Figs. 1 and3). This protein oscillation pattern was also described for mammalian p70S6K (Robles et al. 2017), and for the phosphorylated form of RPS6 which accumulated in the light period especially in polysomes (Turkina et al. 2011; Enganti et al. 2018). Considering that polysome loading associates with plant growth responses (Sulpice et al. 2014) and that S6K promotes translation re-initiation of 5′upstream ORFs, decreasing their ribosome release from these mRNAs, and increasing translation efficiency (Schepetilnikov et al. 2013), we propose that photoperiod regulation of S6K ensures that these energy-consuming processes underlying growth responses are matched to the available resources, especially when hours for photosynthesis are limiting, such as in SDs (Apelt et al. 2017; Henriques et al. 2018).

In agreement with this, we observed that changes in S6K1 levels affect both root and rosette growth under SDs. In fact, over-accumulation of S6K1 had a negative impact on growth that worsened in the absence of circadian regulation (Fig. 4). Similarly, although the deletion of *S6K1* in *s6k1.1* single mutants did not affect root growth, this was not the case when the clock was also affected such as in *ztl-3 s6k1.1* double mutants (Fig. 4). These findings suggest that optimal growth responses require the synchronized presence/activity of several components of the TOR pathway. This gating mechanism, controlled by the clock, would ensure that organ growth patterns are adjusted to specific day lengths and energy levels.

Therefore, any disruptions of the TOR pathway will result in a mismatch between resource generation and utilization with the consequent impact on photoperiod-driven growth. This is the case of TOR-inhibited plants that are unable to mobilize their reserves to adjust to changes in day length (Ren et al. 2012; Dobrenel et al. 2013), as well as *lst8* mutants that fail to adjust their metabolism when moved from SD to LD (Moreau et al. 2012). Moreover, inhibition of the TOR complex results in higher starch levels, has seen in *raptor-1b* mutants that have higher starch content especially under LDs, when their growth patterns are most affected (Caldana et al. 2013; Salem et al. 2018). In contrast, and regardless of photoperiod, *raptor-1b* accumulates lower sucrose and glucose levels, which indicates an impairment in starch degradation in these plants (Artins 2023). However, our metabolic analysis of SD-grown *s6k1.1* mutants differed from *raptor-1b* since starch levels were only slightly elevated at ZT4 and ZT24, but sucrose and glucose accumulation was higher throughout the diel cycle which could be due to altered C partitioning and allocation. Moreover, this metabolic phenotype was reversed in the S6K1-overexpressing line (*S6K1p::S6K1g-CFP#11.6*) which showed significant lower levels of starch, sucrose and glucose during the light period (ZT0-ZT8) (Fig. 5), possibly due to impaired C allocation into growth. This lower starch content during the long night could account for the smaller rosette size of these seedlings (Fig. 4D). Although our findings support previous reports associating the TOR pathway with specific metabolic responses, further research is necessary to understand the specific role of each component. Nevertheless, we show that photoperiod/circadian-dependent regulation of this pathway is critical to ensure that growth responses are matched with the available resources.

The relationship between the circadian clock and the TOR pathway extends from algae to mammals. Rapamycin treatment of 12 L/D grown *Chlamydomonas reinhardtii* cells revealed stronger TOR inhibition in the morning, suggesting a gating mechanism at play (Jüppner et al. 2018). In mice, several components of the oscillator were shown to modulate TOR activity (Jouffe et al. 2013; Khapre et al. 2014a). This circadian regulation promoted the timely accumulation of translation regulators, in an mTOR-dependent manner, to ensure that ribosome biogenesis occurred when resource availability was maximal (Zheng and Sehgal 2010; Wang et al. 2017). Most importantly, direct interaction between Period 2 and mTOR modulated its phosphorylation activity towards S6K, S6 and EBP1, especially during the day when nutrient levels were low (Wu et al. 2019).

In summary, our findings provide further insight on this common regulatory network shared from algae, to plants and animals, where TOR pathway activity would be, at least partially, under circadian control. This would ensure that specific biological processes with high energetic costs occur only when nutrient availability is maximal, especially under short day conditions, when the hours available for photosynthesis are limited. We anticipate that different photoperiods will result in a different S6K accumulation/activity pattern associated with distinct C partitioning and allocation. This dynamic regulation could provide a competitive advantage by allowing organisms to adjust growth patterns to environmental conditions.

## Materials and methods

### Plant material and growth conditions

All the plant material used was in Arabidopsis (*Arabidopsis thaliana*) Col-0 background. The *s6k1-1*, *s6k2-1,* and *s6k2-2* mutants were previously described (Henriques et al. 2010). The *ztl-3* mutant was described before (Jarillo et al. 2001) and the triple *ztl fkf1 lkp2* (Baudry et al. 2010) was kindly provided by Dr Takato Imaizumi. All the transgenic lines generated in this study were evaluated by antibiotic selection and those segregating in conformity with one insertional event were maintained to isolate the corresponding homozygous lines. The ***♀****ztl-3 x **♂** S6K1p::S6K1g-CFP #2.1.3* and *#3.11.4* homozygous lines were generated by crossing the *ztl-3* mutant with the *S6K1p::S6K1g-CFP #11.6* transgenic line, the F1 population was selfed twice and double homozygous were determined by genotyping with specific primers for the *ztl-3* mutation (Supplementary Table S1) and S6K levels were determined by western blot using the anti-S6K antibody (see below; Supplementary Figs. S1 and S2). A similar approach was used to generate the ***♀****ztl-3 x **♂** s6k1.1* double mutant. Briefly, *ztl-3* pistils were pollinated with *s6k1.1* pollen, F1 seeds were tested by PCR to confirm both T-DNA insertions and after selfing for two generations, double homozygous lines were identified. Lack of both *ZTL* and *S6K1* transcripts was detected by RT-qPCR using the primers listed in Supplementary Table S1. Circadian time course experiments were performed either under short day (SD, 8 h light/16 h dark), long day (LD, 16 h light/8 h dark) or free-running conditions where plants were grown under 12 h L/D cycles for 2 weeks and then released in continuous light for 48 h (LL). Unless otherwise stated plants were grown in modified MS medium supplemented with 1% (w/v) of sucrose as described (Kiba and Henriques 2016). To determine rosette area, plants were grown for 21 days under SD conditions in modified MS medium with 1% (w/v) sucrose. Root length determination was done by growing plants vertically under SD conditions in modified MS medium without sucrose. Rosette area and root analysis were done only with seedlings that had developed true leaves (at 21 days) and roots longer than 1 mm (after 10 days). All the other seedlings that failed to develop true rosettes and roots were not included in this analysis. Time course experiments were performed, unless otherwise stated, with 2-week old seedlings. Dissection of roots, cotyledons, and hypocotyls was performed in 2-week-old seedlings grown under the described photoperiods.

### Cloning and generation of transgenic lines

The *S6K1* genomic line C-terminal tagged with CFP were generated by PCR amplification with a proof-reading DNA KOD Hot-start DNA Polymerase (Novagen) and cloned into the pENTRD-TOPO Gateway vector using the manufacturer's protocol and confirmed by sequencing. These clones were then used as templates to generate *AscI-S6K1_pro_:S6K1g(No STOP)-PacI* fragments that were then ligated into the promoterless pBa002a vector to generate *pBa002a/S6K1_pro_:S6K1g-CFP* clones. These were also confirmed by sequencing and used to transform *Agrobacterium* ABI50 strain. Positive colonies were confirmed by PCR and used to transform Col-0 plants. The function of S6K1-CFP in these lines was also confirmed by our team in an independent report (Stitz et al. 2023). In this work, we went further to show that both total and phosphorylated RPS6 levels were not affected in this line (Supplementary Fig. S3I). Primers used in this cloning strategy are listed in Supplementary Table S1.

### Evaluation of expression levels by reverse transcription quantitative PCR

The transcript levels of genes of interest were determined as described (Henriques et al. 2017). Briefly, total RNA was isolated from 2-week old seedlings with the RNeasy Plant RNA Kit and 1 *µ*g of total RNA was used to synthesize cDNA with the AffinityScript qPCR cDNA synthesis kit (Agilent) accordingly to the manufacturer's instructions. RT-qPCR reactions were performed using the SYBR Green Takara qPCR enzyme mix and the results analysed in a LightCycler 480 qPCR machine (Roche). The Ct values obtained with primer pairs amplifying the genes of interest were normalized with *Actin2* using the 2^−ΔCt^ method to provide the relative expression values. All primers used to determine gene expression are listed in Supplementary Table S1.

### Protein detection by western blot and calculation of signal intensities

Protein detection by western blot was done as described previously (Kiba et al. 2007; Henriques et al. 2010). Briefly protein was extracted in 2× SDS-loading dye (SDS-LD) in a ratio of 1:1 (vol buffer: vol of grinded material). Unless otherwise stated, samples were denatured at 95 °C for 5 min and centrifuged for 20 min at 13,000 rpm at 4 °C. In case of phosphatase treatments total protein (60 *µ*g) was extracted in Lacus buffer (Henriques et al. 2010), mixed with 4× SDS-LD and denatured for the same time as described above. A 10% SDS-PAGE gel was used to resolve approximately 60–80 *µ*g of total protein.

Anti-total S6K (Agrisera Antibodies, AS12 1855) and anti-phosphorylated S6K antibodies (Agrisera Antibodies, AS13 2664) were used in a 1:1,000 or 1:750 dilution respectively, in a 7.5% milk TBS-T solution. Anti-total RPS6 (Agrisera Antibodies, AS19 4292) and anti-phosphorylated RPS6 (Ser237) (Agrisera Antibodies, AS19 4291) antibodies were incubated at a 1:1,000 dilution in a 7.5% milk TBT-T solution. Actin was detected using an anti-actin antibody (Agrisera Antibodies, AS13 2640) diluted to 1:5,000 in a 7.5% milk TBS-T solution. Myc-tagged proteins were detected using an anti-Myc (Sigma) antibody at 1:1,000 dilution. Anti-rabbit HRP secondary antibody (for S6K detection) was used at 1:10,000 dilution and anti-mouse HRP secondary antibody was used at a1:5,000 dilution. For chemiluminescence detection, we used the ECL Brilliant bright detection kit (Agrisera Antibodies) and detection was done either in a ImageQuant LAS4000 (GE Healthcare) or the ChemiDoc system (BioRad).

The original (unedited) TIFF files obtained from either the LAS4000 or the ChemiDoc chemiluminescence detection systems were used to determine both signal and background intensity levels for each analysed western blot using the ImageJ program (http://imagej.nih.gov/ij). Briefly for each genetic background/chemical treatment tested we determined the normalized intensity signal of the S6K band. In order to do so, we initially measured the background level of the membrane by determining the signal intensity of all the bands present in the lane being analysed. Then we determined the signal of the S6K band and to this value we subtracted the background value calculated previously. We used the same approach to determine the signal intensity of the loading control bands (actin, tubulin or RuBisco) used in each experiment. Finally, we calculated the ratio between S6K band/loading control band. This value was the S6K normalized amount for each condition/genotype analysed. For time-course experiments, we considered the highest value of S6K accumulation in the wild-type (Col-0, WT) samples which corresponded to ZT3 and was assumed to be 100% to which all the other values obtained were compared. Biological replicates for these experiments are shown in Supplementary Fig. S2. To assess the levels of total and phosphorylated S6K in WT and *ztl-3* mutants, we used the values calculated as described above using the biological duplicates that were normalized with actin in the same membrane. We calculated the signal for S6K and S6K-P assuming that the Col-0 ZT3 sample was 100% (present in all the membranes to allow for normalization).

For experiments with chemical treatments (either CHX or MG132) T0 (corresponds to ZT3, the time of highest S6K accumulation) was considered as 100% and all other values were compared to this. To determine S6K levels in S6K1p::S6K1g-CFP#11.6 lines compared with crossing lines *ztl-3*; S6K1p::S6K1g-CFP#2.1.3 and #3.11.2, we followed a similar approach to determine S6K by normalizing with the loading control band. We then summed up all the values for the time course (ZT0-ZT21) and calculated for each time point what was its % in the total signal. This was calculated for each biological replicate and their average and standard error plotted. Statistically significant differences were calculated using Student's *t* test (*P < 0.05, **P < 0.01) comparing each time point analysed.

### Phosphatase treatments

Total protein was extracted from 10-day old seedlings grown under SD conditions at ZT3 with Lacus buffer supplemented with protease inhibitors (Henriques et al. 2010). 60 *µ*g of total protein were incubated with 60Units of calf intestine alkaline phosphatase (CIAP, Roche) in order to have 1Unit CIAP/*µ*g total protein, in the presence (+) or the absence (−) of phosphatase inhibitors (PIs; 1 mM NaF, 0.5 mM NaVO_3_, 15 mM ß-glycerophosphate and 15 mM pNPP). The reactions were incubated for 1 h at 37 °C and the reaction was stopped by adding 2× SDS-loading dye. In parallel, total protein extracted in Lacus buffer was kept at 4 °C with (+) or without (−) PIs. A separate extraction with 2× SDS-LD was also performed as described before and used as a supplementary control.

### CHX and MG132 treatments

Chemical treatments with either the protein synthesis inhibitor Cycloheximide (CHX), or the proteasome inhibitor MG132 were done as described (Kiba and Henriques 2016). Briefly, 10-day old seedlings were preincubated in liquid medium for several hours and then treated with either 100 *µ*M of either CHX or MG132, and vacuum infiltrated for 10 min. After this, 1 h of incubation was given to allow full entry of chemicals in the cells, this defined T0, which corresponded to ZT3. Incubation was done either in light or dark conditions as described. Protein levels were then analyzed by western blot as described before.

### Determination of root length and rosette leaf area

Seedlings were grown under the conditions described before. Experiments to determine root length were performed with seedlings grown vertically. Root growth and development was followed at day 5, 7, and 10, at this time point photos were taken and root length measured using the Image J program. Rosette area was determined by measuring the rosette areas from photos of seedlings grown under the described conditions. The rosette area was defined by the most outward leaves and calculations were done using the Image J program. Statistical analysis was performed using One-Way ANOVA followed by Tukey post-hoc test (GraphPad Prism Software). Boxplot center lines show the medians; box limits indicate the 10th and 90th percentiles, outliers are represented by dots.

### Metabolite profiling analysis

Arabidopsis plants were grown in soil in a controlled environment chamber under SDs at 20 °C/16 °C under a relative humidity of 75%. After 30 days of growth, whole rosettes were harvested every 4 h. 50 mg of the grounded tissue (*n* = 3 to 5 biological replicates composed by pools of three rosettes) was used for MTBE: methanol:water 3:1:1 (v/v/v) extraction, as described previously (Giavalisco et al. 2011). 150 *μ*l of the organic phase was dried and derivatized as described (Roessner et al. 2001). 1 *μ*l of the derivatized samples were analyzed on an Combi-PAL autosampler (Agilent Technologies GmbH, Waldbronn, Germany) coupled to an Agilent 7890 gas chromatograph coupled to a Leco Pegasus 2 time-of-flight mass spectrometer (LECO, St. Joseph, MI, USA) as described (Weckwerth et al. 2004). Chromatograms were exported from Leco ChromaTOF software (version 3.25) to R software. Peak detection, retention time alignment, and library matching were performed using Target Search R-package (Cuadros-Inostroza et al. 2009). Metabolites were quantified by comparing the integrated peak of a selected mass with a calibration curve obtained using authentic standards. Pairwise comparisons of metabolites between wild-type and mutants at each time point were calculated using Student's *t-*test (*P* < 0.05). For starch quantification, the insoluble material remaining after the MTBE extraction was solubilized in 0.1 M NaOH by heating to 95 °C, neutralized, digested enzymatically overnight and the released glucose was then used to determine starch content of the samples spectrophotometrically by coupling it to the reduction of NADP^+^ to NADPH (Hendriks et al. 2003). The same statistical analysis performed for metabolomics was used for starch content.

### Accession numbers

*Arabidopsis thaliana* accession numbers of protein coding genes used in this work: *S6K1* (At3g08730), *S6K2* (At3g08720), *ZTL* (At5g57360), *LKP2* (At2g18915), and *FKF1* (At1g68050).

## Supplementary Material

kiae254_Supplementary_Data

## Supplementary data

The following materials are available in the online version of this article.

**Supplementary Figure S1**. Characterization of *S6K1* and *S6K2* transcript and protein oscillation patterns in different genetic backgrounds.

**Supplementary Figure S2**. The circadian F-box protein ZTL modulates S6K protein levels (biological replicates used in Fig. 3, A to D).

**Supplementary Figure S3**. *S6K1* and *S6K2* transcript and protein levels show differential accumulation in specific organs.

**Supplementary Table S1**. List of primers.

## Dive Curated Terms

The following phenotypic, genotypic, and functional terms are of significance to the work described in this paper:

TOC1 Gramene: AT5G61380
TOC1 Araport: AT5G61380

AML1 Gramene: AT5G61960
AML1 Araport: AT5G61960

ELF4 Gramene: AT2G40080
ELF4 Araport: AT2G40080

SKP1 Gramene: AT1G75950
SKP1 Araport: AT1G75950

S6K2 Gramene: AT3G08720
S6K2 Araport: AT3G08720

CCA1 Gramene: AT2G46830
CCA1 Araport: AT2G46830

PRR5 Gramene: AT5G24470
PRR5 Araport: AT5G24470

root AmiGo: PO:0009005

ELF3 Gramene: AT2G25930
ELF3 Araport: AT2G25930

ZTL Gramene: AT5G57360
ZTL Araport: AT5G57360

LHY Gramene: AT1G01060
LHY Araport: AT1G01060

metabolite CHEBI: CHEBI:25212
