# Supplementary material for: 40S Ribosomal protein S6 kinase integrates daylength perception and growth regulation in Arabidopsis thaliana
Source: Plant Physiol. 2024 May 3;195(4):3039–52. doi: 10.1093/plphys/kiae254 (PMC11288760; doi:10.1093/plphys/kiae254)
Supplement: kiae254_Supplementary_Data [file kiae254_supplementary_data.zip › Supplementary Data.pdf]

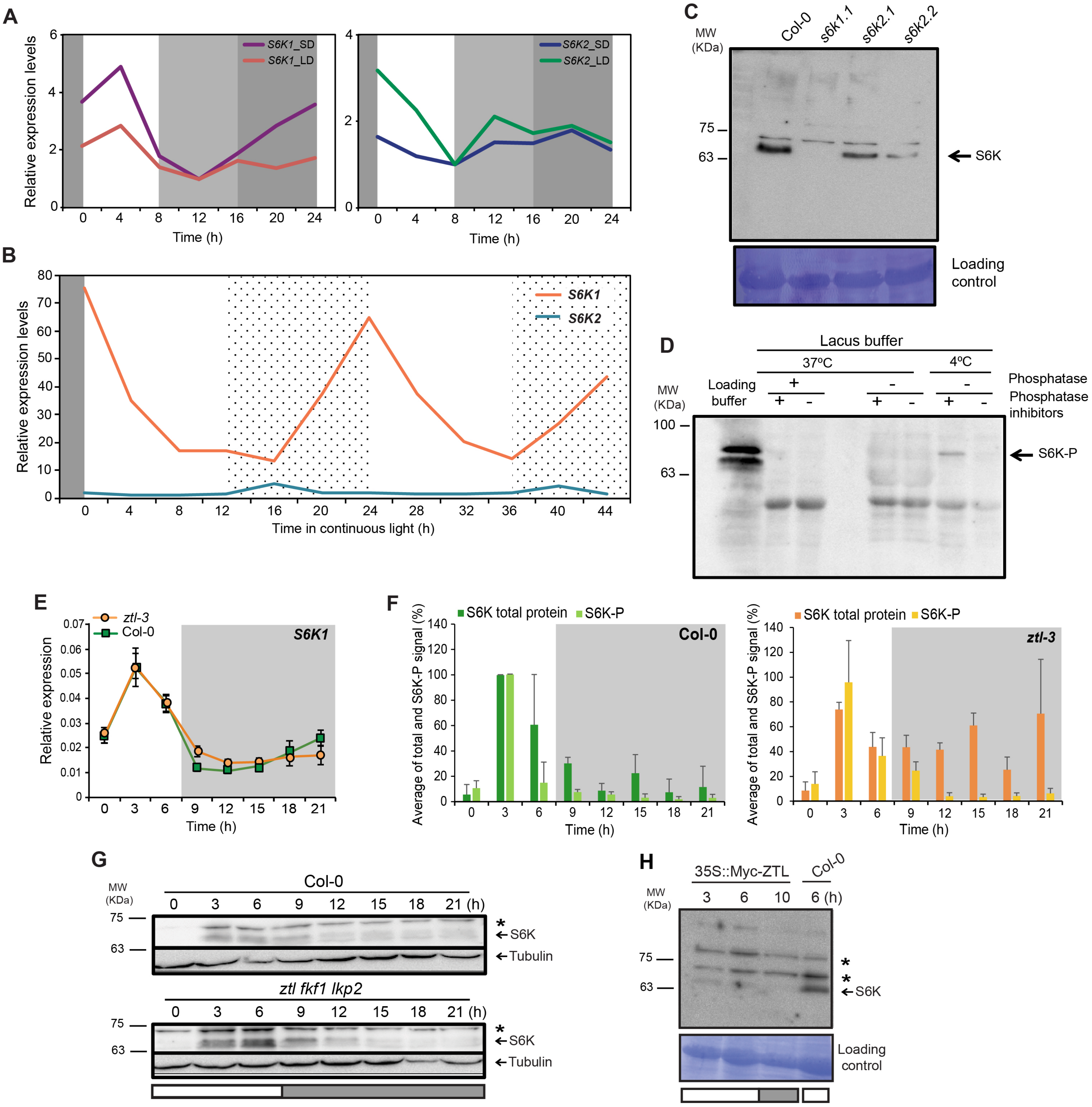

Supplementary Figure S1. Characterization of *S6K1* and *S6K2* transcript and protein oscillation patterns in different genetic backgrounds. (**A**, **B**) Expression data obtained from PHASER (http://phaser.mocklerlab.org) using the settings 'Short Day (SD)', 'Long Day (LD)' and 'LL_LDHC'. *S6K1* and *S6K2* data were obtained searching with 'Array element name 258677_AT' and 'Array element name 258682_AT', respectively. Values were normalized using the lowest 'SD' or 'LD' data as 1, for each transcript described in (**A**); whereas in (**B**) the *S6K2* lowest value was used to normalize both *S6K1* and *S6K2* datasets, to show differences in terms of overall transcript abundance. (**A**) Dark grey rectangles refer to night period under LDs, whereas the combined light and dark rectangles refer to night under SD conditions. (**B**) Dark rectangle refers to night period before release under continuous light and dotted rectangles refer to the subjective night under LL conditions. (**C**) Total S6K protein levels were determined with a specific anti-S6K antibody (S6K; Agrisera Antibodies™) by western blot in wild type (WT, Col-0), *s6k1.1*, *s6k2.1* and *s6k2.2* mutants (upper panel). Loading control was determined by Coomassie staining of the same membrane. The anti-S6K antibody seems to preferably detect S6K1. (**D**) Phosphatase assay to determine specificity of the anti-S6K phosphorylated (S6K-P) antibody. 30 µg of total protein extracted at ZT3 in Lacus buffer was incubated in presence (+) or absence (-) of calf intestine alkaline phosphatase (CIAP), supplemented (+) or not (-) with phosphatase inhibitors (PIs), at 37ºC. Total protein was kept at 4ºC in the presence or absence of PIs (right side of membrane). On first lane total protein was extracted in the presence of 2x SDS-loading dye. Phosphorylated S6K could only be detected either when extracted in 2x SDS-LD or when kept at 4ºC in the presence of PIs without CIAP. Incubation at 37ºC in the absence of PIs was already enough to decrease the levels of S6K-P. Experimental details are given in the Materials and Methods section. Arrows indicate S6K or S6K-P levels. (**E**) *ztl-3* mutants accumulate similar levels of *S6K1* transcripts to WT (Col-0) seedlings. Transcript levels were detected by RT-qPCR. Plants were grown under identical conditions to those used in the western blot analysis. Shown is one representative experiment out of two biological duplicates analyzed. Error bars correspond to ± standard deviation of three technical replicates. (**F**) Comparison between total and phosphorylated S6K levels (after normalising with actin) in Col-0 (left panel) and *ztl-3* mutant (right panel) in two independent biological replicates. Error bars correspond to ± standard deviation. (**G**) Similarly to the *ztl-3* single mutant, two week-old *ztl fkf1 lkp2* triple mutants grown under SD conditions (third panel) accumulated higher S6K protein levels for a longer period of time than WT (first panel). Lower panels (second and fourth) show the loading control determined with the anti-Tubulin (Tubulin) antibody. (**H**) In agreement, two week-old SD-grown ZTL overexpressors (35S::Myc-ZTL) accumulated less S6K (upper panel). Coomassie staining of the same membrane shows loading of samples (lower panel). Arrows indicate S6K or Tubulin levels, asterisk points to unspecific band. (**E-H**) White and grey rectangles represent light and dark periods, respectively. Time after lights on is shown as (h). MW refers to protein molecular weight marker bands in KDa.

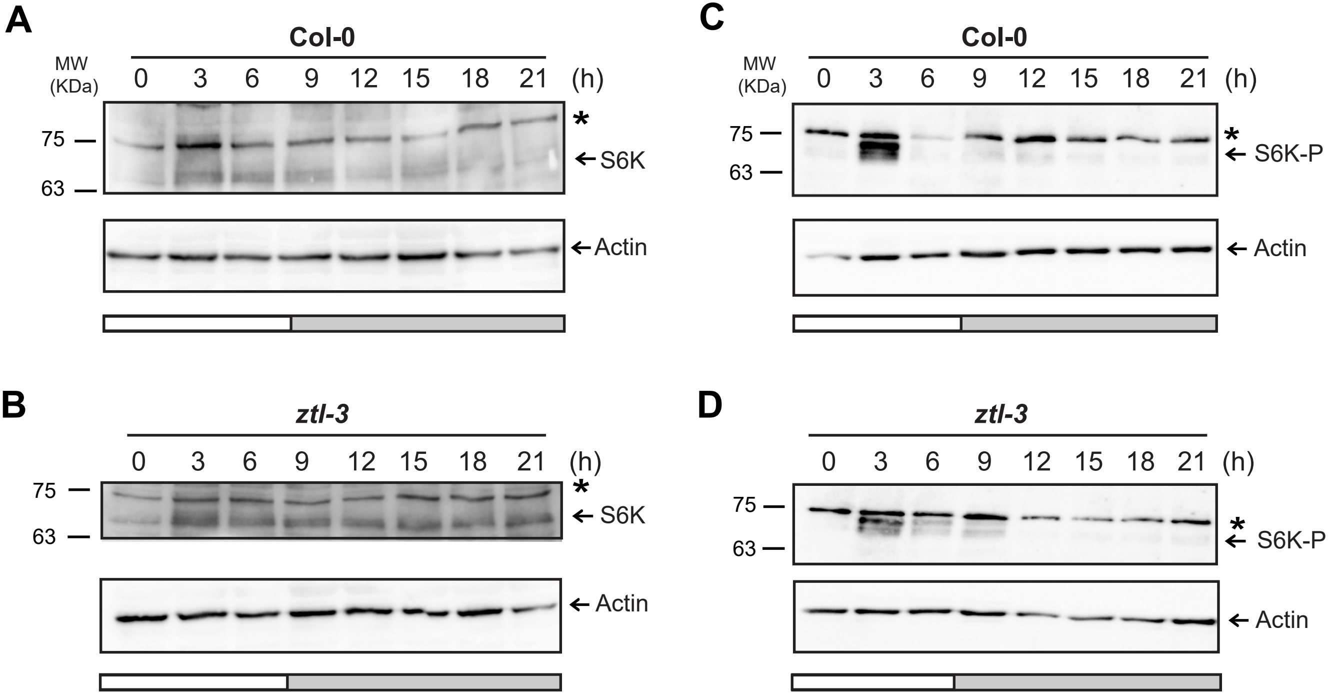

Supplementary Figure S2. The circadian F-box protein ZTL modulates S6K protein levels (biological replicates used in Figure 3A-D). Total S6K (S6K) and actin protein levels in WT plants (**A**) and *ztl-3* mutants (**B**) were detected by western blot analysis using anti-S6K and anti-actin specific antibodies. Arrows point to S6K and actin bands, respectively. Star indicates an unspecific band. (**C**) Phosphorylated S6K (S6K-P) levels were detected in WT seedlings and *ztl-3* mutants (**D**) using anti-S6K-P and anti-actin specific antibodies. Loading control was determined by actin levels in the same blots. Arrows point to S6K-P and actin bands, respectively. Star indicates an unspecific band. MW refers to protein molecular weight marker bands in KDa. Time refers to hours (h) after lights on; white and grey rectangles indicate the light and dark periods, respectively.

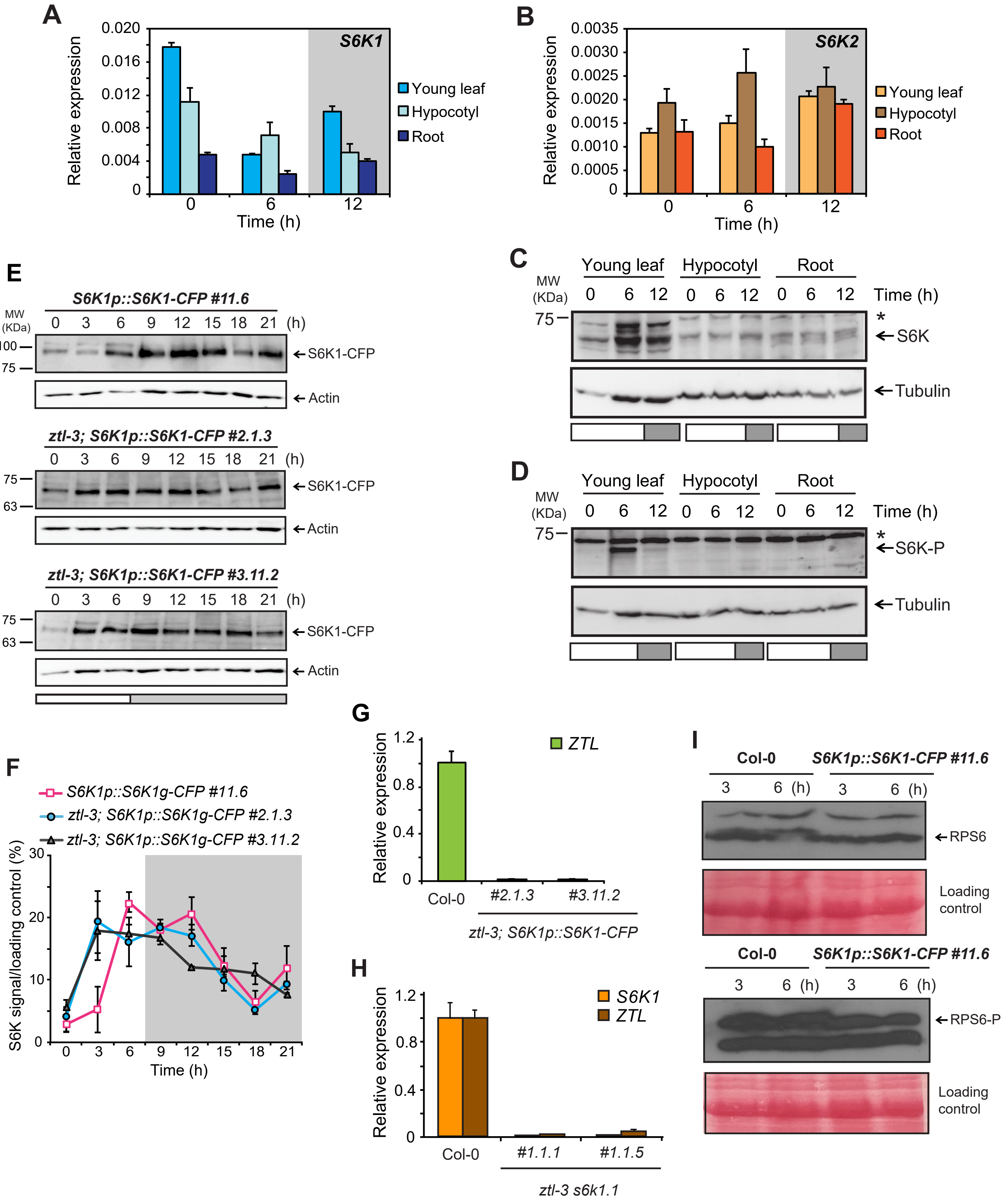

Supplementary Figure S3. *S6K1* and *S6K2* transcript and protein levels show differential accumulation in specific organs. Ten day-old seedlings grown under SD conditions were dissected in order to separate root, hypocotyl and cotyledon, which were used to determine *S6K1* (**A**) and *S6K2* (**B**) transcript levels. Transcript levels were evaluated by RT-qPCR normalized with *Actin2* in three technical replicates. Shown is one representative experiment out of two biological duplicates analysed. (**C**) The same plant material used in (**A**) and (**B**) was also tested for total S6K protein accumulation (upper panel). Total protein levels were determined using an anti-tubulin antibody (lower panel). (**D**) Similarly, phosphorylated S6K levels (S6K-P) were determined in the same set of samples (upper panel), loading control was determined by incubation with anti-tubulin antibody. Arrows indicate S6K, S6K-P or Tubulin levels, asterisk points to unspecific band. (**E**) Crossing of the *S6K1*-genomic line *S6K1p::S6K1g-CFP*#11.6 to *ztl-3* mutants lead to a slight increase in S6K1-CFP protein at ZT0-ZT3, as seen in two independent lines (middle and lower panels) when compared with one of the parents (upper panel) grown under SDs. Loading controls were determined using an anti-actin antibody. Arrows point to S6K1-CFP and actin bands, respectively. (**F**) Average signal intensity of S6K1 after normalizing with the loading control in two independent biological duplicates. (**A-F**) Day and night are indicated by white and grey rectangles, respectively. (**G**) Absence of *ZTL* transcripts in *ztl-3; S6K1p::S6K1g-CFP* #11.6 crosses #2.13 and 3.11.2 was evaluated by RT-qPCR normalized with *Actin2* in three technical replicates. (**H**) Absence of *S6K1* and *ZTL* transcripts in *ztl-3 s6k1.1* crosses #1.1.1 and #1.1.5 was evaluated as described in (**G**). (**I**) Total RPS6 (top panels) and RPS6 phosphorylated levels (lower panels) at ZT3 and ZT6 were similar in wild type (Col-0) and in the *S6K1p::S6K1g-CFP* #11.6 line, confirming that the CFP tag did not impair S6K1 function. Loading controls were determined by Ponceau staining of the same membranes. Arrows point to RPS6 and RPS6-P bands, respectively. (**A**, **B**, **F-H**) Error bars refer to standard deviation values. (**C-E**) MW refers to protein molecular weight marker bands in KDa. Time after lights on is shown as (h).

Supplementary Table S1. List of primers.

1. Cloning of S6K1 genomic fragments into pBa002a

Primer name	Sequence (5’ – 3’)
S6K1g-AscI-Fw	GCCGGCGCGCCAAAATCAGCAAATTCATACAAGAACT
S6K1g-PacI-Rev	GCCTTAATTAACAAAGTAGTTGTGGACTGGTGAAG

2. Genotyping of *ztl-3* mutants

Primer name	Sequence (5’-3’)
ZTL-Fw	TACACAGTATTCACCGAGTCCT
ZTL-Rev	TTCTCTTTCATACATAGGCTCTC
T-DNA	GCGTGGACCGCTTGCTGCAACT

3. Evaluation of gene expression by qPCR

Primer name	Sequence (5’ – 3’)
S6K1-q1-F	GGAGATCTGGTGAAAGTCTCAGGTGTAGTA
S6K1-q1-R	AAGATACAGCCTGTACTTGGTCTGAAAAGA
S6K2-q1-R	GCTTCAGGCATTGGTCCAAAGACAT
S6K2-q1-F	ACCAGACTGGGAAGCCGTTTCAGA
ZTL-3'UTR-Fw	GTGCTTGTTTCTGTTGTTCTCTTT
ZTL-3'UTR-Rev	GCATAAGAAGAGAAAGGATCTTATTA
