# Supplementary material for: 40S Ribosomal protein S6 kinase integrates daylength perception and growth regulation in Arabidopsis thaliana
Source: Plant Physiol. 2024 May 3;195(4):3039–52. doi: 10.1093/plphys/kiae254 (PMC11288760; doi:10.1093/plphys/kiae254)
Supplement: kiae254_Supplementary_Data [file pp.01659.2023-s03.pdf › pp.01659.2023-s03.pdf]

Plant Physiology®

40S Ribosomal Protein S6 Kinase (S6K1) Integrates Day Length Perception And Growth Regulation In Arabidopsis

Marc Boix, Alba Garcia, Laia Castillo, Bernat Miro, Ferga Hamilton, Sanata Tolak, Adrian Perez, Carolina Monte-Bello, Camila Caldana, and Rossana Henriques

Review Timeline:

Submission Date:	03-Aug-2023
Editorial Decision:	04-Sep-2023
Resubmission Received:	31-Oct-2023
Editorial Decision:	18-Nov-2023
Revision Received:	26-Feb-2024
Editorial Decision:	12-Mar-2024
Revision Received:	29-Mar-2024
Accepted:	09-Apr-2024

04-Sep-2023

Dr. Rossana Henriques
University College Cork
Cork
Ireland

RE: 40S Ribosomal Protein S6 Kinase (S6K1) Integrates Day Length Perception And Growth Regulation In Arabidopsis

Dear Dr. Rossana Henriques:

Thank you for giving us the opportunity to review your manuscript for publication in the Plant Physiology. The reviewers have found the manuscript of interest, but have raised a number of concerns that will require your attention. As these concerns are of some gravity and will entail substantial changes to the manuscript, you are likely to need additional time to address them. I must therefore reject the manuscript in its present form. Nonetheless, if you are able to address the points raised in review, I would welcome your resubmission of a manuscript that incorporates your responses.

Please note that any resubmission will be treated as a new entry to the system and is likely to go out for review.

If you do have any queries or concerns, you are welcome to contact me in the first instance.

Thank you again for allowing Plant Physiology to review your work. We look forward to hearing from you soon.

Sincerely,

Board of Editors Plant Physiology

-- Reviewer comments:
Reviewer #1 (Comments for the Author):

The manuscript from Boix et al., reports evidences of the involvement of the kinase S6K1 in day-length perception in Arabidopsis. They present data on circadian regulation of S6K1, involvement of an F-box protein in this process, and also evidences that the amount of S6K1 protein affects both plant growth and sugars accumulation.
Overall, this manuscript is well written, present relevant data of good quality and is of general interest for the plant science community and probably beyond knowing the conservation of S6Ks in eukaryotes. However, several important revisions should be made (particularly major revisions 1 and 2).

MAJOR REVISIONS:
Major revision 1: The authors sometimes refer to TOR involvement in S6K activation (lines 174; 340-341 and in Fig. 5C). but most data rely on S6K amount and when S6K phosphorylation is studied, it is measured relative to loading control, but not to S6K amount which fluctuate a lot. Hence, if we compare Fig 1C and 1D, there is a strong possibility that S6K-P signal only increases because S6K global signal increases, but not because "TOR complex activity towards S6k is maximal at specific times of the day". A similar comment also concerns the sentence line 340-341 "phosphorylated S6K was stabilized in ztl-3 mutant.", but Fig 3B suggests that the stabilization concern globally S6K, not particularly the phosphorylated form. Analysis of the S6K-P/S6K ratio should be made to reach conclusions regarding the involvement of TOR.

Major revision 2. The S6Kp::S6K1-CFP lines are used as overexpressor lines and are named "S6K1 overexpressor line" only in the discussion (line401). I recognize the interest to use S6K1 promoter for this purpose instead of a constitutive promoter that would also lead to missexpression. However, It would be better to introduce the fact that these lines are overexpressor lines earlier in the manuscript (in paragraph pp248-257). Also, at the same time, the authors should provide evidences that the S6K1-CFP fusion protein is functional. It is important, because both figures 4 and 5 depend on this assumption. Indeed, expression of S6K1-CFP represses growth (Figure 4), a result that could be coherent with the possibility that the fusion with CFP would lead to an inactive version of the kinase. This could be addressed by either complementation of an S6k1 mutant phenotype (maybe possible in the ztl-3 background), or by evidences that an S6K1 target, like S6, is phosphorylated by S6K1-CFP. An alternative could be to show similar data than Fig 4 and 5 with unfused S6K1 (S6K1p::S6K1g).

MINOR REVISIONS:
Minor revision 1: it would be useful to show the full pictures of the Western blots, at least for Supp Fig 1.C and D showing how the S6K and S6K-P bands are identified in regard to the background. Also, the replicated of each Western blot should be shown in supplementary data to allow the readers/reviewers to fully analyze these data.

Minor revision 2: line 254-255. The different effect of ztl-3 background on S6K1-CFP (supp Fig 2F) compared to endogenous S6K1 (Fig 3B) should be discussed.

Minor revision 3:
-Line 443. Is it not F1 instead of M1 as the sentence is about a crossing? If not, the sentence is confusing.

Minor revision 4:
-Line206-208. (Fig 3D) This sentence « S6K-P levels were higher from ZT3 to ZT9..." concerns a non-significative difference (if not, the statistical analysis is not clear). It should be mentioned.

Minor revision 5:
It would be nice to give a reference regarding the sentence lines163-164 as the information found on the antibody provider's website (Agrisera) is not very clear.

Reviewer #2 (Comments for the Author):

This paper explores the regulation of the expression of the S6 kinase, part of the energy-sensing TOR kinase pathway that has been implicated in the regulation of protein synthesis as well as cell cycle control. The S6 kinase has multiple targets in cell cycle regulation and the translation apparatus, and mutants in it have very intriguing genome integrity defects. This paper makes novel and largely conclusive contributions to our understanding of this important yet understudied regulatory kinase.

The data indicate that the S6K protein is highly unstable, and the mRNA and protein levels are regulated by photoperiod, the circadian clock and light and darkness. There is also evidence from multiple experiments that S6K protein turnover is regulated by the clock protein ZTL, that S6K1 and S6K2 isoforms vary substantially in their mode of regulation and that S6K is also under translational control. Finally, comparing s6k1 mutants and S6K overexpressors, S6K level affects diel cycles of sugar levels, (glc, frc and suc). The authors identified a second (tagged) S6K transgene that is misregulated. This transgene appears to cause a synthetic enhanced growth defect when combined with the clock mutation ztl, another indicator that the levels of this kinase need to be carefully calibrated.

The conclusions appear to be generally supported by the data shown, with one major exception as I will point out.

Comments:

Abstract:
1. 'S6K1 sits at the intersection of TOR and circadian perception' sounds potentially confusing given that S6K is downstream of TOR. Would it be more precise to say, 'S6K appears to be a nexus where metabolic signaling by the TOR pathway is modulated by circadian timing'?
2. The word 'perfect' at the end seems a bit subjective.
3. 'when C/energy levels are limiting then changes in S6K1 levels affect starch ... '. But according to the data in Fig. 5A-B sugars are affected less in darkness, when C/energy is limiting, but more in the light when C /energy levels are still high; starch is barely affected (also see line 122).

Line 137. I think you don't want to refer to Fig. 1 here, yet.
Line 146. When WT seedlings were entrained... (add 'were').
Line 148. It appears that the cycle under cLL does not peak at dawn but peaks during the subjective night, clearly phase-advanced as compared to LD. The RT-PCR results seem to underscore this phase shift as compared to the public array data in Fig. S1, which showed a smaller shift. Regardless, both data show the shift. This is quite interesting given that the phosphorylation of S6K's target protein, RPS6, follows a very similar pattern, a morning peak in LD but a subjective-night peak in cLL (Chaudhary 2015; Enganti 2018).
Line 172. It seems clear that S6K cycles less under long-day, but I would not describe it as (completely) lost, but rather 'reduced'.

Figure 1.
The S6K protein appears to run at different molecular weights in different gels. In Fig. 1 it runs at about 53kDa as expected from its molecular weight. But in Fig. S1 panels C, D, F, G it runs at 68kDa. But it is this 68kDa band that is absent in the s6k1 mutant, strong evidence it is the correct band.
Figure 1.
In the legend the dark gray and light gray night periods are not described accurately.

Figure 2 and 3
These experiments show quite clearly that S6K levels are suppressed by ZTL but not other clock-related ZTL-like F-box proteins. They also show that S6K is indeed a very unstable protein, and its stability and half life is regulated by ZTL.

Line 254.
In Figure S2, the authors asked whether the regulation by ZTL can be detected for a transgenic S6K1-CFP fusion protein. Surprisingly, the fusion protein behaves quite different from the wild type, which the authors do not comment on. The fusion protein does not disappear in the night as the WT does, and ZTL does not affect S6K-CFP levels at night. The result in Fig. S2 should still be shown, but it needs a bit more context and discussion. Example: ZTL only affects S6K-CFP in the morning, which seems surprising given that ZTL is an evening-gene. I suspect that ZTL fails to degrade the S6K1-CFP over several hours at night, and the cumulative effect of this becomes evident only in the morning.

Line 252. In Fig. S2 the S6Kp:S6K-CFP OE construct is in the homozygous ztl mutant background. Therefore the genotype should be denoted as ztl; S6Kp:S6K-CFP, not as ztl x S6K... The x leads us to believe that the plants are F1 hybrids.

Figure 4. Why so much variation in the root length assay? Maybe this is because sucrose was omitted from the growth medium (line 459). Perhaps adding some sucrose would make results more uniform.

Line 283 and line 398. Authors state that starch is elevated in s6k1 mutants (Fig. 5A). But the figure does not bear this out. Starch is completely normal in s6k1. Other sugars are elevated however. This interpretation of this result should be corrected.

In line 295 the authors probably mean to say 'rosette growth' rather than seedling growth, since it is rosettes that are affected by S6K-OE (Fig. 4D).

Figure 5.
It seems odd that the variance in the data for WT differs a lot between figure panels A and B. In (A) the variance looks what one might expect for this type of assay while in (B) the variance is extremely small. If the SD values do not capture the actual variation in the data, then the statistical tests will be exaggerating differences between genotypes. In fact we can tell that the variance is higher than shown in (B), because panel (A) shows Glc and Frc values that are only about half of those in (B) for the same genotype (WT).

Answers to reviewers

We would like to thank both reviewers for their helpful comments which contributed to improve this final version of our manuscript. All answers go below each comment, in blue. We would like to mention that our references to the numbers of lines refer to the word file where we have kept the track changes tool.

Reviewer #1 (Comments for the Author):

The manuscript from Boix et al., reports evidences of the involvement of the kinase S6K1 in day-length perception in Arabidopsis. They present data on circadian regulation of S6K1, involvement of an F-box protein in this process, and also evidences that the amount of S6K1 protein affects both plant growth and sugars accumulation.
Overall, this manuscript is well written, present relevant data of good quality and is of general interest for the plant science community and probably beyond knowing the conservation of S6Ks in eukaryotes. However, several important revisions should be made (particularly major revisions 1 and 2).

MAJOR REVISIONS:

Major revision 1: The authors sometimes refer to TOR involvement in S6K activation (lines 174; 340-341 and in Fig. 5C). But most data rely on S6K amount and when S6K phosphorylation is studied, it is measured relative to loading control, but not to S6K amount which fluctuate a lot. Hence, if we compare Fig 1C and 1D, there is a strong possibility that S6K-P signal only increases because S6K global signal increases, but not because "TOR complex activity towards S6k is maximal at specific times of the day". A similar comment also concerns the sentence line 340-341 "phosphorylated S6K was stabilized in *ztl-3* mutant.", but Fig 3B suggests that the stabilization concern globally S6K, not particularly the phosphorylated form. Analysis of the S6K-P/S6K ratio should be made to reach conclusions regarding the involvement of TOR.

The reviewer raises a relevant point and we did a complementary analysis of our results with two biological replicates (Col-0 and *ztl-3* mutant seedlings) that were used in Figure 3. This strategy was devised due to the fact that all these westerns had a similar sample (Col-0 ZT3) and all these membranes were tested with the same anti-actin antibody, so we could compare these experiments using a similar strategy where the Col-0 ZT3 signal (either S6K or S6K-P) is considered 100%. So, this allowed us to compare, within each genotype, the values for S6K and S6K-P signals. We also included a description of these calculations in the Material and Methods section (lines 529-534). This analysis is now included in Supplementary Figure 1F and it shows that in Col-0 indeed the accumulation of S6K-P matches the accumulation of total S6K protein. However, in the *ztl-3* mutant we saw that though total S6K accumulation is extended even during the night period, the level of phosphorylated S6K is only extended for 3h, until ZT9 (3h after lights off). We interpret this data as TOR phosphorylation occurring mostly during the light period, and we hypothesise that this post-translational modification could also signal for proteasome degradation of S6K. In our understanding these results do suggest that TOR will phosphorylate S6K mostly during the light period and not the dark, even if S6K total levels are present. We have, nevertheless, rewritten our text to reflect in a more accurate manner these findings, this can be seen in lines (174, 205-216, 341, 349-351). In addition, we also updated the legend in Figure 5C (line 936).

Major revision 2. The S6Kp::S6K1-CFP lines are used as overexpressor lines and are named "S6K1 overexpressor line" only in the discussion (line401). I recognize the interest to use S6K1 promoter for this purpose instead of a constitutive promoter that would also lead to missexpression. However, It would be better to introduce the fact that these lines are overexpressor lines earlier in the manuscript (in paragraph pp248-257).

We clarified this point in the manuscript (lines 256-258).

Also, at the same time, the authors should provide evidences that the S6K1-CFP fusion protein is functional. It is important, because both figures 4 and 5 depend on this assumption. Indeed, expression of S6K1-CFP represses growth (Figure 4), a result that could be coherent with the possibility that the fusion with CFP would lead to an inactive version of the kinase. This could be addressed by either complementation of an S6k1 mutant phenotype (maybe possible in the ztl-3 background), or by evidences that an S6K1 target, like S6, is phosphorylated by S6K1-CFP. An alternative could be to show similar data than Fig 4 and 5 with unfused S6K1 (S6K1p::S6K1g).

The *S6Kp::S6K1-CFP* line has been functionally confirmed in a separate publication where the role of the TOR pathway in lateral root development was tested (Figure 3D; Stitz et al, 2023; https://doi.org/10.15252/embj.2022111273). In this report we and our collaborators show that S6K1-CFP can be detected in the early stages of lateral root development where it overlaps with Raptor 1b accumulation and is involved in promoting this process. We believe these findings are good evidence for the functionality of our line and we included this information in the main text (lines 257-258) and also in the material and methods section (lines 481-482).

MINOR REVISIONS:

Minor revision 1:

it would be useful to show the full pictures of the Western blots, at least for Supp Fig 1.C and D showing how the S6K and S6K-P bands are identified in regard to the background. Also, the replicated of each Western blot should be shown in supplementary data to allow the readers/reviewers to fully analyze these data.

We have changed Supplemental Fig 1C and 1D to include full length membranes, to show that the signal corresponding to S6K1 is clear in relation to the background. We have a very big number of biological duplicate and triplicate westerns, therefore we selected a few biological replicates corresponding to Figure 3A-D where we have the data relating to S6K and S6K-P accumulation in WT and *ztl-3* seedlings and created a new supplemental figure (Supplemental Figure 2) to include these results. We then updated the text to reflect that we now have 3 supplemental figures.

Minor revision 2:

-line 254-255. The different effect of *ztl-3* background on S6K1-CFP (supp Fig 2F) compared to endogenous S6K1 (Fig 3B) should be discussed.

A similar question was raised by reviewer 2 and we introduced some more detail on the description of S6K1-CFP accumulation in relation to the wild type S6K1 levels. We hypothesise that in the S6K1g-CFP line the levels of S6K1 during the night might not be fully degraded by wild type levels of ZTL. This results in higher S6K1g-CFP during most of the diel cycle, which will be increased in *ztl-3* mutants leading to extra accumulation in the morning. We also included this explanation in the text (lines 262-265).

Minor revision 3:

-Line 443. Is it not F1 instead of M1 as the sentence is about a crossing? If not, the sentence is confusing.

We corrected this designation throughout the text (lines 450, 455).

Minor revision 4:

-Line206-208. (Fig 3D) This sentence « S6K-P levels were higher from ZT3 to ZT9..." concerns a non-significative difference (if not, the statistical analysis is not clear). It should be mentioned.
We corrected this sentence in the text (lines 213-216).

Minor revision 5:

It would be nice to give a reference regarding the sentence lines163-164 as the information found on the antibody provider's website (Agrisera) is not very clear.

We used here the information given by Agrisera, to our knowledge this is the first report using this antibody, so we can’t provide a reference.

Reviewer #2 (Comments for the Author):

This paper explores the regulation of the expression of the S6 kinase, part of the energy-sensing TOR kinase pathway that has been implicated in the regulation of protein synthesis as well as cell cycle control. The S6 kinase has multiple targets in cell cycle regulation and the translation apparatus, and mutants in it have very intriguing genome integrity defects. This paper makes novel and largely conclusive contributions to our understanding of this important yet understudied regulatory kinase.

The data indicate that the S6K protein is highly unstable, and the mRNA and protein levels are regulated by photoperiod, the circadian clock and light and darkness. There is also evidence from multiple experiments that S6K protein turnover is regulated by the clock protein ZTL, that S6K1 and S6K2 isoforms vary substantially in their mode of regulation and that S6K is also under translational control. Finally, comparing s6k1 mutants and S6K overexpressors, S6K level affects diel cycles of sugar levels, (glc, frc and suc). The authors identified a second (tagged) S6K transgene that is misregulated. This transgene appears to cause a synthetic enhanced growth defect when combined

with the clock mutation ztl, another indicator that the levels of this kinase need to be carefully calibrated.

The conclusions appear to be generally supported by the data shown, with one major exception as I will point out.

Comments:

Abstract:

1. 'S6K1 sits at the intersection of TOR and circadian perception' sounds potentially confusing given that S6K is downstream of TOR. Would it be more precise to say, 'S6K appears to be a nexus where metabolic signaling by the TOR pathway is modulated by circadian timing'?

We re-wrote the abstract to address this point, although we think our results indicate that S6K would be a hub where the phosphorylation of TOR and regulation by the circadian clock do converge (lines 47-48).

2. The word 'perfect' at the end seems a bit subjective.

We re-wrote this sentence to incorporate this comment (lines 52-53).

3. 'when C/energy levels are limiting then changes in S6K1 levels affect starch ... '. But according to the data in Fig. 5A-B sugars are affected less in darkness, when C/energy is limiting, but more in the light when C /energy levels are still high; starch is barely affected (also see line 122).

This sentence relates to the SD photoperiods which we analysed in more detail, and not so much to the dark/light changes in a 24h period, we clarified this in line 48. Concerning line 122, we corrected this sentence to include "sugars" since we have observed changes in their levels, especially in the S6K1-CFP Ox line.

Line 137. I think you don't want to refer to Fig. 1 here, yet.

We clarified this aspect in the text (lines 136-137) since this first reference to *S6K2* was to the Diurnal database, so we just maintained the reference to Supplemental Figure 1A and B.

Line 146. When WT seedlings were entrained... (add 'were').

This was corrected in the manuscript text. Thank you for spotting it.

Line 148. It appears that the cycle under cLL does not peak at dawn but peaks during the subjective night, clearly phase-advanced as compared to LD. The RT-PCR results seem to underscore this phase shift as compared to the public array data in Fig. S1, which showed a smaller shift. Regardless, both data show the shift. This is quite interesting given that the phosphorylation of S6K's target protein,

RPS6, follows a very similar pattern, a morning peak in LD but a subjective-night peak in cLL (Chaudhary 2015; Enganti 2018).

This is an interesting point with which we agree. We changed the text to reflect this aspect (line 148), which as highlighted by the reviewer, is also in agreement with what has been described for RPS6. Concerning the relevance of this phase advance and seasonal perception, our results combined with those of Chaudhary and Enganti do offer new perspectives on the relevance of circadian regulation of S6K.

Line 172. It seems clear that S6K cycles less under long-day, but I would not describe it as (completely) lost, but rather 'reduced'.

We agree with this comment and changed the text accordingly (line 171).

Figure 1.

The S6K protein appears to run at different molecular weights in different gels. In Fig. 1 it runs at about 53kDa as expected from its molecular weight. But in Fig. S1 panels C, D, F, G it runs at 68kDa. But it is this 68kDa band that is absent in the s6k1 mutant, strong evidence it is the correct band.

We checked the MW marker labelling in our figures since we used two different brands in these figures, we are certain to be identifying the same band since in these westerns we always included an *s6k1.1* mutant sample. We have updated this information in Figure 1.

Figure 1.

In the legend the dark gray and light gray night periods are not described accurately.

We corrected this in the text, thank you for spotting it.

Figure 2 and 3

These experiments show quite clearly that S6K levels are suppressed by ZTL but not other clock-related ZTL-like F-box proteins. They also show that S6K is indeed a very unstable protein, and its stability and half life is regulated by ZTL.

We thank the reviewer for this comment.

Line 254.

In Figure S2, the authors asked whether the regulation by ZTL can be detected for a transgenic S6K1-CFP fusion protein. Surprisingly, the fusion protein behaves quite different from the wild type, which the authors do not comment on. The fusion protein does not disappear in the night as the WT does, and ZTL does not affect S6K-CFP levels at night. The result in Fig. S2 should still be shown, but it needs a bit more context and discussion. Example: ZTL only affects S6K-CFP in the morning, which

seems surprising given that ZTL is an evening-gene. I suspect that ZTL fails to degrade the S6K1-CFP over several hours at night, and the cumulative effect of this becomes evident only in the morning.

This is a relevant point, which was also raised by reviewer 1 and we have included an explanation in the text to address this comment (lines 262-265).

Line 252. In Fig. S2 the S6Kp:S6K-CFP OE construct is in the homozygous ztl mutant background. Therefore the genotype should be denoted as ztl; S6Kp:S6K-CFP, not as ztl x S6K... The x leads us to believe that the plants are F1 hybrids.

We corrected this description throughout the manuscript.

Figure 4. Why so much variation in the root length assay? Maybe this is because sucrose was omitted from the growth medium (line 459). Perhaps adding some sucrose would make results more uniform.

We used standard protocols to assess root phenotypes and these do not include sucrose in the media. Adding sucrose will prevent roots from growing straight and that also affects results.

Line 283 and line 398. Authors state that starch is elevated in *s6k1* mutants (Fig. 5A). But the figure does not bear this out. Starch is completely normal in *s6k1*. Other sugars are elevated however. This interpretation of this result should be corrected.

We found a small, but significant, increase in starch content in *s6k1.1* at ZT4, nevertheless we corrected the text to include this information (lines 293-294).

In line 295 the authors probably mean to say 'rosette growth' rather than seedling growth, since it is rosettes that are affected by S6K-OE (Fig. 4D).

We corrected this sentence to reflect the phenotype we describe in Figure 4 (line 306).

Figure 5.

It seems odd that the variance in the data for WT differs a lot between figure panels A and B. In (A) the variance looks what one might expect for this type of assay while in (B) the variance is extremely small. If the SD values do not capture the actual variation in the data, then the statistical tests will be exaggerating differences between genotypes. In fact we can tell that the variance is higher than shown in (B), because panel (A) shows Glc and Frc values that are only about half of those in (B) for the same genotype (WT).

We would like to clarify that panel A and panel B correspond to different experiments which were done in parallel, due to technical reasons we could not evaluate all the samples together. We did evaluate several biological replicates and we are aware that there is some experimental variation. We observed that sugar levels in wild type (WT) oscillate within a range of values and there isn't an absolute value for those plants. However, we did observe consistently the same relationship

between WT and mutants (*s6k1.1*) which accumulated more sugars, whereas the overexpressor line (S6K1-CFP) accumulated less sugar (with the exception of fructose especially at ZT8). Below we present a summary of our results, where we analysed the different genotypes in two batches (batch 2 and batch 6)

				**µmol g-1 FW**	**nmol g-1 FW**		
**Batch**	**Photoperiod**	**Time**	**Genotype**	**Starch**	**Sucrose**	**Fructose**	**Glucose**
2	SD	0	wt	5.9	622.6	97.1	209.9
2	SD	0	wt	7.9	720.3	66.3	185.1
2	SD	0	wt	5.9	626.5	87.7	252.1
2	SD	4	wt	36.2	1858.2	581.8	1570.5
2	SD	4	wt	34.8	1655.7	720.8	1469.0
2	SD	4	wt	35.2	1632.3	627.1	1484.6
2	SD	8	wt	55.0	1718.4	284.4	954.3
2	SD	8	wt	59.1	1747.9	223.1	816.0
2	SD	8	wt	57.0	1829.1	227.7	888.9
2	SD	12	wt	49.1	1057.2	61.9	177.8
2	SD	12	wt	41.5	1261.2	102.1	203.6
2	SD	12	wt	44.4	992.7	153.7	289.9
2	SD	16	wt	30.3	1096.0	73.9	199.5
2	SD	16	wt	31.6	1071.5	123.1	272.3
2	SD	16	wt	29.1	1053.1	178.6	318.5
2	SD	20	wt	21.3	987.1	108.0	279.0
2	SD	20	wt	18.8	940.9	84.4	235.5
2	SD	20	wt	19.6	991.8	94.3	245.4
2	SD	24	wt	7.9	679.0	107.9	288.6
2	SD	24	wt	7.3	616.6	82.5	246.0
2	SD	24	wt	7.9	579.9	71.1	182.1
2	SD	0	S6K1_OX	4.3	554.4	71.4	181.1
2	SD	0	S6K1_OX	5.6	502.1	86.9	237.5
2	SD	0	S6K1_OX	5.0	395.2	42.1	93.5
2	SD	4	S6K1_OX	27.4	1277.5	574.8	1109.2
2	SD	4	S6K1_OX	29.8	1328.8	601.8	1208.7
2	SD	4	S6K1_OX	27.7	1177.2	491.4	995.2
2	SD	8	S6K1_OX	43.1	1551.8	483.1	968.1
2	SD	8	S6K1_OX	46.8	1468.3	561.0	1072.8
2	SD	8	S6K1_OX	46.0	1640.9	558.5	1093.5
2	SD	12	S6K1_OX	39.5	1219.3	295.3	439.0
2	SD	12	S6K1_OX	38.1	1141.3	110.1	233.5
2	SD	12	S6K1_OX	40.5	1153.0	138.1	257.7
2	SD	16	S6K1_OX	29.1	1056.6	132.4	262.2
2	SD	16	S6K1_OX	32.8	1044.1	118.9	248.6

2	SD	16	S6K1_OX	30.5	987.1	87.8	160.0
2	SD	20	S6K1_OX	9.6	686.1	132.9	234.0
2	SD	20	S6K1_OX	13.1	877.0	244.0	475.7
2	SD	20	S6K1_OX	15.1	1352.5	175.1	407.3
2	SD	24	S6K1_OX	5.2	555.9	92.9	195.6
2	SD	24	S6K1_OX	5.3	534.2	73.4	175.0
2	SD	24	S6K1_OX	5.1	484.0	131.3	235.2
				**µmol g-1 FW**	**nmol g-1 FW**		
**Batch**	**Photoperiod**	**Time**	**Genotype**	**Starch**	**Sucrose**	**Fructose**	**Glucose**
6	SD	0	wt	4.9	339.7	61.4	196.0
6	SD	0	wt	4.7	393.6	56.3	235.4
6	SD	0	wt	5.0	507.3	98.8	199.8
6	SD	0	wt	5.3	356.0	57.3	126.4
6	SD	0	wt	5.2	403.8	42.1	209.4
6	SD	4	wt	34.4	576.4	267.6	634.6
6	SD	4	wt	29.7	1023.0	599.4	1156.4
6	SD	4	wt	32.3	554.7	323.0	720.2
6	SD	4	wt	33.0	790.1	343.3	765.2
6	SD	4	wt	31.3	1002.7	543.2	974.0
6	SD	8	wt	55.7	1060.9	213.7	617.9
6	SD	8	wt	57.3	1351.4	255.9	720.1
6	SD	8	wt	53.5	1146.3	195.0	582.1
6	SD	8	wt	51.8	1304.5	220.2	754.1
6	SD	8	wt	51.8	633.8	145.4	337.7
6	SD	12	wt	41.9	1288.5	176.4	327.6
6	SD	12	wt	47.9	1098.2	136.1	252.1
6	SD	12	wt	40.9	1032.1	184.5	269.2
6	SD	12	wt	39.6	393.4	52.3	96.0
6	SD	12	wt	45.7	828.1	146.3	297.6
6	SD	16	wt	33.5	1175.7	139.9	350.0
6	SD	16	wt	34.3	845.5	136.4	252.3
6	SD	16	wt	35.1	870.6	104.7	189.3
6	SD	16	wt	33.7	825.5	84.2	182.2
6	SD	16	wt	32.6	549.7	88.9	160.5
6	SD	20	wt	18.6	740.6	104.2	210.4
6	SD	20	wt	18.2	743.3	145.7	277.4
6	SD	20	wt	17.8	656.9	104.2	299.7
6	SD	20	wt	16.5	740.1	135.8	389.0
6	SD	20	wt	17.7	704.2	128.5	304.2
6	SD	24	wt	7.4	746.4	146.5	326.2
6	SD	24	wt	8.4	643.5	128.6	281.8
6	SD	24	wt	7.9	497.5	124.4	319.0
6	SD	24	wt	8.4	488.4	120.4	310.2
6	SD	24	wt	7.8	676.5	220.0	416.2

6	SD	0	s6k1.1	6.0	469.9	74.2	172.7
6	SD	0	s6k1.1	5.8	481.5	79.0	196.0
6	SD	0	s6k1.1	5.7	571.5	194.0	374.9
6	SD	0	s6k1.1	5.1	660.5	140.7	299.7
6	SD	0	s6k1.1	4.6	534.7	174.4	456.0
6	SD	4	s6k1.1	36.7	1311.3	885.5	1644.2
6	SD	4	s6k1.1	36.6	1604.3	915.2	1894.4
6	SD	4	s6k1.1	39.4	1287.7	715.8	1503.2
6	SD	4	s6k1.1	35.5	1325.8	681.6	1716.4
6	SD	4	s6k1.1	33.8	1071.9	545.7	1238.9
6	SD	8	s6k1.1	62.1	742.3	216.9	426.8
6	SD	8	s6k1.1	53.3	1176.1	292.3	755.5
6	SD	8	s6k1.1	55.9	1074.2	223.3	603.2
6	SD	8	s6k1.1	51.4	2072.0	1030.6	2194.0
6	SD	8	s6k1.1	56.4	1622.0	629.4	1338.3
6	SD	12	s6k1.1	42.7	971.9	237.5	339.6
6	SD	12	s6k1.1	45.4	1005.6	106.9	272.2
6	SD	12	s6k1.1	44.8	1029.2	96.5	251.4
6	SD	12	s6k1.1	45.4	1107.6	184.0	303.3
6	SD	12	s6k1.1	48.7	1123.3	161.3	342.3
6	SD	16	s6k1.1	34.1	942.1	187.2	378.8
6	SD	16	s6k1.1	33.7	1056.9	241.3	541.1
6	SD	16	s6k1.1	33.1	1009.8	164.7	294.4
6	SD	16	s6k1.1	33.6	1140.3	197.6	371.9
6	SD	16	s6k1.1	36.3	1123.7	111.0	216.9
6	SD	20	s6k1.1	18.3	1360.2	490.8	815.5
6	SD	20	s6k1.1	18.6	912.4	160.7	295.8
6	SD	20	s6k1.1	18.5	962.7	219.3	381.0
6	SD	20	s6k1.1	16.5	781.4	145.1	327.9
6	SD	20	s6k1.1	21.1	982.3	239.8	389.3
6	SD	24	s6k1.1	6.5	404.4	86.7	227.0
6	SD	24	s6k1.1	5.9	442.6	67.6	194.0
6	SD	24	s6k1.1	5.7	546.8	107.6	205.1
6	SD	24	s6k1.1	6.3	450.7	85.7	272.1
6	SD	24	s6k1.1	6.2	534.0	90.1	183.7

18-Nov-2023

Dr. Rossana Henriques
University College Cork
Cork
Ireland

RE: 40S Ribosomal Protein S6 Kinase (S6K1) Integrates Day Length Perception And Growth Regulation In Arabidopsis

Dear Dr. Henriques:

We have received reviews of your manuscript, which you may view at the end of this letter. On the basis of the advice received and my own evaluation, I believe that, pending revision, this article may ultimately be acceptable for publication in Plant Physiology.

Please provide evidence that the S6K1-CFP fusion protein is functional as this is actually very important (see reviewer 1). Moreover, please address the other comments and suggestions of the reviewers.

When you are ready to submit your revision, click the below link and find the red arrow under your Author Tasks heading for this revision submission.

If you want to visit this version of the manuscript, you may click the link below and then click on the folder that reads "Post Decision Manuscripts".

Link Not Available

When uploading the revised version of this article, please be sure to include as complete an answer as possible to the referees' criticisms during the first round of reviews in the "response to reviewers" section.

Plant Physiology considers a revision for up to 60 days after a manuscript has been provisionally accepted; limited extension of this timeframe is at the discretion of the Monitoring Editor. A reminder notice will be sent to you 2 weeks before the deadline, at which time you may choose to contact the editorial office to request an extension; if so, please provide a justification for your request. After the expiration of this deadline, the article would need to be submitted as a resubmission and would be handled as such.

Thank you very much for giving us an opportunity to review this work. I look forward to receiving the next version.

Sincerely,

Board of Editors Plant Physiology

Want to add this revision deadline to your calendar? Click below!

-- Reviewer comments:
Reviewer #1 (Comments for the Author):

As mentionned in my first review, I think that this work is of great interest for the plant science community. In this revised manuscript, the authors have done some nice improvements, particularly regarding my major comment 1.

However, they did not reply appropriately to my second major comment asking for evidences that the S6K1-CFP fusion protein is functional (I clearly mentioned the possibility that the fusion with CFP would lead to an inactive version of the kinase). There reply mention previously published data indicating that the S6K1-CFP protein is expressed in lateral roots but these data do not address the question of the functionality of the fused S6K1. Indeed, the new sentence "where S6K1 is overexpressed under the

control of its own promoter and shown to be active in lateral root formation (Stitz et al., 2023)" (line 258) is misleading because it was not shown that S6K1 is active. Therefore, this issue is still a problem and none of the suggested experiments to resolve it were tested.

Reviewer #2 (Comments for the Author):

The authors edited the paper according to reviewers' recommendations. I have only a few additional suggestions:

Line 81: delete the word 'matches'.
Line 108: Is Khapre an appropriate reference here given it refers to mouse data? Khapre does not seem to have circadian data, only diel data.
Line 124: Should 'circadian' be replaced by 'diel' or 'rhythmic'?
Line 263-265: Change tense from future/present tense to past tense as in the rest of the results.
Line 268: Double mutant genotypes should not be denoted with 'x'. This is unconventional and confusing. Instead of stl- x s6k1.1 it should be ztl-3 s6k1-1. This also applies elsewhere in the manuscript.
Line 340: Two words are crossed out. Please edit as needed.

Answers to reviewers

We would like to thank both reviewers for their helpful comments. All answers go below each comment, in blue. We would like to mention that our references to the numbers of lines refer to the word file where we have kept the track changes tool.

Reviewer #1 (Comments for the Author):

As mentioned in my first review, I think that this work is of great interest for the plant science community. In this revised manuscript, the authors have done some nice improvements, particularly regarding my major comment 1.

However, they did not reply appropriately to my second major comment asking for evidences that the S6K1-CFP fusion protein is functional (I clearly mentioned the possibility that the fusion with CFP would lead to an inactive version of the kinase). There reply mention previously published data indicating that the S6K1-CFP protein is expressed in lateral roots but these data do not address the question of the functionality of the fused S6K1. Indeed, the new sentence "where S6K1 is overexpressed under the control of its own promoter and shown to be active in lateral root formation (Stitz et al., 2023)" (line 258) is misleading because it was not shown that S6K1 is active. Therefore, this issue is still a problem and none of the suggested experiments to resolve it were tested.

We tested, using western blot, both the levels of total and phosphorylated ribosomal S6 protein and added these results to Supplemental Fig 3I. As seen in that figure, both wild type (Col-0) and the S6K1-CFP line show comparable levels of total and phosphorylated S6 protein (Ser237), the conserved target of S6K1, at ZT3 and ZT6. In this experiment we used 10 day old seedlings grown under SD conditions which were in all similar to all our previous experiments. We included this information in lines 259-262 and also in lines 514-516, we also updated the figure legends.

We believe these results clearly show that the S6K1-CFP accumulation in these lines is still reflected in the phosphorylation of S6. Moreover, we would also like to emphasize that our metabolic data already pointed out that S6K1-CFP accumulation would be functional, since the metabolic phenotype of these seedlings was mostly the opposite of that of the *s6k1.1* mutant (Fig 5).

Reviewer #2 (Comments for the Author):

The authors edited the paper according to reviewers' recommendations. I have only a few additional suggestions:

Line 81: delete the word 'matches'.

We did it change (line 82 in this revised version).

Line 108: Is Khapre an appropriate reference here given it refers to mouse data? Khapre does not seem to have circadian data, only diel data.

This reference refers to the connection between the mouse metabolic clock and the TOR signalling pathway. Since in this paragraph we refer to TOR both in plants and animals, we think it would still make sense to use this reference. However, the reviewer does raise a good point in terms of the diel regulation, and therefore we included “*diel and*” in line 108 to reflect this.

Line 124: Should 'circadian' be replaced by 'diel' or 'rhythmic'?

In line 126 we replaced the word ‘circadian’ with ‘diel’.

Line 263-265: Change tense from future/present tense to past tense as in the rest of the results.

This was corrected as suggested (lines 269-270).

Line 268: Double mutant genotypes should not be denoted with 'x'. This is unconventional and confusing. Instead of *ztl-3 x s6k1.1* it should be *ztl-3 s6k1-1*. This also applies elsewhere in the manuscript.

We corrected this in the manuscript and also in all the figures and corresponding figure legends.

Line 340: Two words are crossed out. Please edit as needed.

We could not find this in our own version, so we assumed it might have been corrected when we saved the most recent version of the document.

12-Mar-2024

Dr. Rossana Henriques
University College Cork
Cork
Ireland

RE: 40S Ribosomal Protein S6 Kinase (S6K1) Integrates Day Length Perception And Growth Regulation In Arabidopsis

Dear Dr. Rossana Henriques:

We are pleased to accept your manuscript for publication in the Plant Physiology. This acceptance is contingent on revision based on the minor comments of our reviewers.

Please highlight all changes and include a detailed annotation to changes to the text, with line numbers, and noting your responses to the comments.

To submit your revised manuscript, click:

Link Not Available

If you cannot return the revised manuscript within 8 weeks of receipt, please let us know. Otherwise, we will assume that you have elected not to revise the manuscript and are withdrawing it.

Thank you for allowing us to review your work. We look forward to hearing from you soon.

Sincerely,

Board of Editors, Plant Physiology

-- Reviewer comments:
Reviewer #1 (Comments for the Author):

The new data on S6 phosphorylation in the S6K1p::S6K1g-CFP line is interesting and informative. Even if it doesn't demonstrate overactivation of S6K in plants overexpressing CFP tagged S6K1 (this might be difficult to detect), it shows that the fused protein do not decrease S6K activity as we could expect id the fused protein was inactivated.
However, the interpretation needs clarification:
- Line 261 to 262; according to Material and Methods, the transformation was done in plants containing WT S6Ks (Col-0 WT) (line 491). Therefore, the sentence "We confirmed that the CFP tag did not affect S6K1 accumulation nor its ability to phosphorylate the ribosomal protein S6 (RPS6)" is confusing because we don't know if the S6K activity observed is due to the endogenous S6Ks or to the S6K-CFP fused S6K. This should be clarified, e.g. "nor the ability of endogenous S6Ks or fused S6K1 to phosphorylate the ribosomal protein S6 (RPS6)".
The following sentence taken from the authors's response to reviewers should also be added to the manuscript as it would also help clarify the situation "both wild type (Col-0) and the S6K1-CFP line show comparable levels of total and phosphorylated S6 protein (Ser237)"

Also, Line263. As mentioned previously, S6K activity was not measured in the cited work, therefore "active in" should be replaced by "expressed during". or the authors should indicate that the verb "active" correspond to CFP here, not to S6K1

Reviewer #2 (Comments for the Author):

I do not have any further comments.

Answers to reviewers

All answers go below each comment, in blue. We would like to mention that our references to the numbers of lines refer to the word file where we have kept the track changes tool.

Reviewer #1 (Comments for the Author):

The new data on S6 phosphorylation in the S6K1p::S6K1g-CFP line is interesting and informative. Even if it doesn't demonstrate overactivation of S6K in plants overexpressing CFP tagged S6K1 (this might be difficult to detect), it shows that the fused protein do not decrease S6K activity as we could expect id the fused protein was inactivated.

We understand this, but we think that it might not be straight forward to find an increase in RPS6 phosphorylation, probably it just stays for longer under SD conditions. This would be something we will test in the future, for this work we chose ZT3 and ZT6 because these are the time points when S6K accumulation is maximal in wild type seedlings and we wanted to show that no major difference is found.

However, the interpretation needs clarification:

- Line 261 to 262; according to Material and Methods, the transformation was done in plants containing WT S6Ks (Col-0 WT) (line 491). Therefore, the sentence "We confirmed that the CFP tag did not affect S6K1 accumulation nor its ability to phosphorylate the ribosomal protein S6 (RPS6)" is confusing because we don't know if the S6K activity observed is due to the endogenous S6Ks or to the S6K-CFP fused S6K. This should be clarified, e.g. "nor the ability of endogenous S6Ks or fused S6K1 to phosphorylate the ribosomal protein S6 (RPS6)".

We corrected this sentence (now in line 260-261).

- The following sentence taken from the authors's response to reviewers should also be added to the manuscript as it would also help clarify the situation "both wild type (Col-0) and the S6K1-CFP line show comparable levels of total and phosphorylated S6 protein (Ser237)"

We added this information in lines 262-263.

Also, Line263. As mentioned previously, S6K activity was not measured in the cited work, therefore "active in" should be replaced by "expressed during" or the authors should indicate that the verb "active" correspond to CFP here, not to S6K1

We replaced “active in” with “expressed during” now (line 264).

Reviewer #2 (Comments for the Author):

I do not have any further comments.

09-Apr-2024
Dr. Rossana Henriques
University College Cork
School of Biological, Earth and Environmental Sciences
Cork
Ireland

MSID: PP2023-RA-01659DR2
MS TITLE: 40S Ribosomal Protein S6 Kinase (S6K1) Integrates Day Length Perception And Growth Regulation In Arabidopsis

Dear Dr. Rossana Henriques:

I am pleased to inform you that your revised manuscript "40S Ribosomal Protein S6 Kinase (S6K1) Integrates Day Length Perception And Growth Regulation In Arabidopsis" has been accepted for publication in Plant Physiology. Your manuscript will be checked for consistency with journal formatting and image standards by one of our Science Editors, who will contact you shortly. They may have a number of queries and suggestions for you. Please keep in mind that they work to increase the online visibility of your article and its attraction for readers from the widest of backgrounds. You will have the opportunity to review any revisions, and I hope you will work constructively with them to get the most out of your research. You will also have the opportunity to review all changes when you receive the author proofs for your article.

Once validated for publication, your article will be sent to a Science Editor for a final review of your manuscript and source files. After your final files are uploaded, your article will appear in the next available issue and the online Preview version of your article will be posted after your signed Copyright and Checklist forms and final source files are received.

Please be sure to include the following in your manuscript file:
Harvard style citations
List of all author contributions and funding information at the beginning of manuscript after the titles and author list.
List all supplemental materials associated with the article, with titles, at the end of the ms, before the references and acknowledgments section.
Complete Figure Legends for all figures appearing in the article listed before the Literature Cited.

Thank you and congratulations on your Plant Physiology paper!

Sincerely,
Board of Editors Plant Physiology

=====
FIRST AUTHOR PROFILES
=====

We recognize the hard work that goes into being First Author of a Plant Physiology paper by publishing a "First Author Feature" for each First Author alongside your paper.

If the first author(s) would like to submit information for a feature on Facebook and Twitter, fill out the form by clicking on the link below:

Plant Physiology First Author Feature Form: https://forms.office.com/r/ZBCGZfhm8F
If you cannot access the form through the link above, please contact Rachel at rbelsky@aspb.org.

To upload an image of yourself to accompany your profile, please put your photo in the correct journal folder at the link below. You must label your image: Author First Name Last Name Article Title.

First Author Profile Image Dropbox: https://www.dropbox.com/scl/fo/xz029p7qjr2jml2rp2kmq/h?dl=0&rlkey=6fkxcaqmxx35ap152b2osy1fz

=====

IMPORTANT REMINDER: PEER REVIEW REPORTS
=====

If you opted to publish a peer review report along with your article during the original submission process, it will be prepared by the editorial staff and publicly posted with your manuscript, inside the zip file that contains any other supplemental material. As a reminder, the peer review report is a public record of all comments from editors and reviewers, as well as your prior responses, as you received them in the decision letters for each draft of your manuscript. If you agreed to publish this report and have changed your mind, or are not sure if you selected this option, please contact the editorial office as soon as possible before signing the license agreement from our publisher.

-- Reviewer comments:
Reviewer #1 (Comments for the Author):

I think the correction made are appropriate and I do not have any further comments

Reviewer #2 (Comments for the Author):

I do not have any further comments.
